# Endothelial Filamin C Alleviates Atherosclerosis via PINK1/Parkin-Dependent Mitophagy and mtDNA-cGAS-STING Inflammation Suppression

**DOI:** 10.3390/jcdd13090428

**Published:** 2026-09-01

**Authors:** Yongxuan Zhan, Yan Feng, Ting Zhao, Jiapeng Fu, Minghui Chen, Mingli Liu, Yu Cao, Jiaju Li, Xiangqi Meng, Yongli Li

**Affiliations:** 1 Department of Neurosurgery, The Second Clinical Medical College, Harbin Medical University, Harbin 150086, China; zhanyongxuan@hrbmu.edu.cn (Y.Z.);; 2 Key Laboratory of Myocardial Ischemia, Ministry of Education, Harbin 150086, China

**Keywords:** atherosclerosis, FLNC, mitophagy, PINK1/Parkin pathway, cGAS-STING pathway

## Abstract

Endothelial dysfunction and impaired mitophagy represent core pathological drivers of atherosclerosis progression, yet upstream molecular regulators governing this process in vascular endothelium remain largely uncharacterized. This study delineated the functional role and mechanistic basis of filamin C (FLNC) in atherosclerosis-related endothelial injury. We profiled FLNC expression in 20 paired human carotid plaque and adjacent normal vascular tissues collected from patients undergoing carotid endarterectomy. In vitro, CRISPRa/CRISPRi-stable HUVEC lines were challenged with ox-LDL, with pharmacological (Mdivi-1) and genetic (si-PINK1/si-Parkin) rescue assays used to verify causal signaling. In vivo, Tie2-driven endothelial-specific FLNC-overexpressing ApoE^−/−^ mice were fed a high-fat diet to induce atherosclerotic lesions. FLNC was significantly downregulated in human plaques, ox-LDL-treated HUVECs, and atherosclerotic mouse aortas. FLNC overexpression attenuated endothelial apoptosis and oxidative stress, while its knockdown exacerbated these injuries. Mechanistically, FLNC activated PINK1/Parkin-mediated mitophagy to restrict cytosolic mtDNA leakage and suppress subsequent activation of the cGAS-STING inflammatory cascade. Mitophagy inhibition or PINK1/Parkin silencing fully abrogated FLNC’s cytoprotection, and endothelial FLNC overexpression retarded plaque progression in vivo. Collectively, our findings identify FLNC as a novel endogenous endothelial mitophagy regulator, revealing an unreported regulatory mechanism and supporting FLNC as a promising therapeutic target for atherosclerotic disease.

## 1. Introduction

Atherosclerosis-related cardiovascular and cerebrovascular diseases are the predominant factors responsible for global deaths, which imposes immense pressure on public health governance worldwide [1,2]. As the physiological barrier of the vascular wall, the vascular endothelium is susceptible to dysfunction, which represents the initiating event of atherosclerosis and persists throughout the entire process of plaque initiation, progression, destabilization and rupture [3,4]. Endothelial apoptosis, dysregulated oxidative stress, and excessive inflammatory activation collectively impair endothelial barrier function, promote lipid deposition and monocyte infiltration, and ultimately drive plaque formation and progression [5,6]. Therefore, identifying endogenous protective factors that maintain endothelial homeostasis and inhibit endothelial injury is of critical clinical significance for the early prevention and treatment of atherosclerosis.

Endothelial energy turnover and intracellular redox stability rely heavily on the functional activity of mitochondria. Mitochondrial dysfunction is an early key event in endothelial injury [7,8]. Mitophagy, the primary mitochondrial quality control mechanism, selectively removes damaged mitochondria via the autophagolysosomal system to maintain cellular homeostasis [9,10]. The PINK1/Parkin signaling pathway is the canonical mitophagy pathway; its dysfunction causes massive accumulation of damaged mitochondria, leading to mitochondrial reactive oxygen species (mtROS) burst, membrane potential decrease, and apoptosis [11,12]. In atherosclerosis, impaired PINK1/Parkin-mediated mitophagy exacerbates endothelial oxidative stress and dysfunction [13,14]. Recent studies show that mitochondrial DNA (mtDNA) released from damaged mitochondria acts as a signal, activating the cytosolic cGAS-STING pathway to trigger inflammatory cascades that drive atherosclerosis [15,16]. However, upstream endogenous regulators of this PINK1/Parkin-mtDNA-cGAS-STING axis in endothelial cells remain poorly defined, limiting the development of mitochondria-targeted atherosclerosis therapies.

Filamin C (FLNC), one actin-binding protein belonging to the filamin family, is predominantly expressed in striated muscles, where it cross-links actin filaments to maintain cytoskeletal stability [17,18]. Loss-of-function FLNC mutations are closely correlated with muscular dystrophies, hypertrophic cardiomyopathy, and other myopathies [19,20]. Our preliminary data show that FLNC is basally expressed in vascular endothelial cells, but its biological functions in vascular physiology and pathology remain completely unknown. Emerging studies indicate that actin-binding proteins also participate in intracellular signal transduction and organelle homeostasis beyond cytoskeletal maintenance [19,21], suggesting that FLNC may have non-canonical functions in endothelial cells. Given the central role of mitophagy in endothelial protection against atherosclerosis, we hypothesize that FLNC may maintain endothelial mitochondrial homeostasis and inhibit mtDNA-cGAS-STING pathway activation by regulating PINK1/Parkin-mediated mitophagy, thereby exerting endothelial protective and anti-atherosclerotic effects.

Given the above background, both cellular and animal experimental systems were adopted to dissect FLNC’s functional impacts on atherosclerosis-triggered endothelial impairment. We also unraveled the underlying molecular cascade whereby FLNC governs mitophagic activity and endothelial inflammatory signaling. The present research seeks to lay fresh theoretical groundwork and identify promising molecular markers for developing endothelium-focused strategies to prevent and treat atherosclerosis.

## 2. Materials and Methods

### 2.1. Ethics Statement

Ethical review and approval for collecting human clinical samples were granted by the Ethics Board of the Second Clinical Medical College of Harbin Medical University (Ethics Approval No. YJSKY2024-395), and all involved patients delivered signed informed consent forms. Every animal experiment implemented in this work adhered rigorously to the official Guide for the Care and Use of Laboratory Animals, and the entire animal study protocol was authorized by the same institutional Ethics Committee (Ethics Approval No. YJSDW2024-283).

### 2.2. Clinical Sample Collection

Twenty carotid artery specimens were harvested from patients receiving carotid endarterectomy at the Second Clinical Medical College of Harbin Medical University from May 2024 to November 2024. For each specimen, plaque tissue and matched normal vascular tissue from the patent distal end of the same vessel were isolated, rapidly frozen in liquid nitrogen, and stored at −80 °C for subsequent protein extraction.

### 2.3. Cell Culture and Stable Cell Line Construction

HUVECs (ZQ1099, ZQXZBIO, Shanghai, China) were cultured in endothelial cell-specific medium (ZM1099, ZQXZBIO) at 37 °C in a humidified atmosphere with 5% carbon dioxide mixed with 95% ambient air. All cellular assays were carried out with cells at fewer than five passages.

Human aortic endothelial cells (HAECs, ZQY001, ZQXZBIO, Shanghai, China) were cultured in endothelial cell-specific complete medium (ZMY001, ZQXZBIO, Shanghai, China) under the same temperature and gas atmosphere conditions as HUVECs. HAECs were used for the validation of key experimental findings via transient transfection, and all HAEC-related assays were performed with cells at fewer than five passages.

Stable cell lines with endogenous activation (CRISPRa/VP64 activation system, VB240801-1282awq, VB240812-1685suv, VB010000-9384wwc, VB010000-9383ffr, Vector Builder, Guangzhou, China) and inhibition (CRISPRi/KRAB repression system, VB240801-1301xnw, VB240812-1683rjn, VB010000-9386mwf, Vector Builder, Guangzhou, China) of FLNC were constructed using lentiviruses, along with corresponding empty vector control stable lines [22]. All lentiviruses were purchased from Vector Builder (Guangzhou, China). Cells were plated into six-well culture dishes and infected using the corresponding lentiviruses (MOI = 15) plus 5 μg/mL polybrene when cell confluence reached 50%. After 12 h of incubation, the supernatant was removed and replaced with fresh culture medium. After another 48 h of culture, cells were selected with antibiotic for 2 weeks. We confirmed the transfection efficiency through Western blotting alongside immunofluorescence before subsequent experiments.

### 2.4. Cell Grouping and Treatment

An endothelial injury model was established by treatment with 25 μg/mL ox-LDL (CSP136, ZQXZBIO) for 24 h, which was applied consistently in both HUVECs and HAECs. HUVECs were used for complete mechanistic dissection, while HAECs were used for independent validation of the core findings. Oxidized low-density lipoprotein (ox-LDL)-induced injury in human umbilical vein endothelial cells (HUVECs) is a widely recognized classic in vitro model for the study of early-stage atherosclerotic endothelial dysfunction, which aligns well with the scientific focus of the present study. The construction protocol of this model was developed with reference to published well-established experimental procedures [23]. The experiments were divided into the following modules:

Bidirectional functional verification module (in both HUVECs and HAECs): ① Control (con) group: Untreated cells cultured in normal medium. ② ox-LDL group: Cells treated with 25 μg/mL ox-LDL for 24 h. ③ FLNC overexpression (OE) group: Stable cells transduced with CRISPRa-FLNC vector and treated with 25 μg/mL ox-LDL for 24 h. ④ Overexpression negative control (OE-NC) group: Stable cells transduced with CRISPRa scrambled gRNA vector, treated with 25 μg/mL ox-LDL for 24 h. ⑤ FLNC knockdown (KD-FLNC) group: Stable cells transduced with CRISPRi-FLNC vector, treated with 25 μg/mL ox-LDL for 24 h. ⑥ Knockdown negative control (KD-NC) group: Stable cells transduced with CRISPRi scrambled gRNA vector, treated with 25 μg/mL ox-LDL for 24 h.

Mitophagy inhibition rescue module (in HUVECs): ① con group; ② ox-LDL group; ③ OE group; ④ OE+DMSO group: FLNC stable cells treated with 25 μg/mL ox-LDL and DMSO for 24 h; ⑤ OE+Mdivi-1 group: FLNC stable cells treated with 10 μmol/L Mdivi-1 (HY-15886, MCE, Monmouth Junction, NJ, USA) and 25 μg/mL ox-LDL for 24 h [24,25].

PINK1/Parkin silencing rescue module (in both HUVECs and HAECs): ① con group; ② ox-LDL group; ③ OE group; ④ OE+si-Scramble group: OE cells transfected with non-silencing control siRNA; ⑤ OE+si-PINK1 group: OE cells transfected with PINK1-specific siRNA; ⑥ OE+si-Parkin group: OE cells transfected with Parkin-specific siRNA. Except for the con group, all other groups were stimulated with 25 μg/mL ox-LDL for 24 h. siRNAs were synthesized by GenePharma. Cells were transfected with si-PINK1 (seq: 5′-GAAGCCACCAUGCCUACAUTT-3′) or si-Parkin (seq: 5′-CUGCCGGGAAUGUAAAGAATT-3′) at a final concentration of 100 nM with siRNA-mate Plus transfection reagent (GenePharma, Shanghai, China). Transfection efficiency was verified by Western blotting 48 h after transfection before different cell treatments.

### 2.5. Western Blot Assay

Total protein from cell pellets and tissues was extracted with RIPA lysis buffer (G2002, Sevier Biotechnology, Wuhan, China). BCA reagent (P0010S, Beyotime, Shanghai, China) was utilized to measure the concentration of total protein. Equal amounts of protein samples were resolved by 6–12% SDS-polyacrylamide gel electrophoresis (SDS-PAGE) (PG120, PG121, PG122, PG123, Epyzime, Shanghai, China), followed by electrotransfer onto 0.45 μm polyvinylidene difluoride (PVDF) membranes (FFP106, Beyotime) at a constant current of 400 mA. Membranes were blocked with blocking solution for 1 h at room temperature.

Membranes were subjected to primary antibody incubation at 4 °C throughout the night: FLNC (1:1200, A13018, ABclonal, Wuhan, China), PINK1 (1:1200, A7131, ABclonal), Parkin (1:1200, A0968, ABclonal), p62 (1:1200, A19700, ABclonal), LC3B (1:1200, A19665, ABclonal), TOM20 (1:1500, AF1717, Beyotime), cleaved-caspase-3 (1:5000, 68773-1-Ig, Proteintech, Wuhan, China), p-TBK1 (1:1200, AP1026, ABclonal), TBK1 (1:4000, A3458, ABclonal), p-STING (1:1200, AP1223, ABclonal), STING (1:2000, A21051, ABclonal), p-IRF3 (1:1200, AP0995, ABclonal), IRF3 (1:11000, A27272, ABclonal), TNF-α (1:10000, A22227, ABclonal), MCP-1 (1:11000, 29547-1-AP, Proteintech), TFAM (1:4000, A23467, ABclonal), CD31 (1:2000, 28083-1-AP, Proteintech), α-SMA (1:20000, 67735-1-Ig, Proteintech), eNOS (1:800, A20985, ABclonal), p-eNOS (1:800, AP1404, ABclonal), β-actin (1:100000, AC026, ABclonal), vinculin (1:320000, A2752, ABclonal). After five washes with TBST (5 min for every cycle), the membranes underwent incubation with horseradish peroxidase-conjugated secondary antibodies (goat anti-rabbit immunoglobulin, 1:10000, AS014, ABclonal; goat anti-mouse immunoglobulin, 1:5000, RGAM001, Proteintech) for 1 h at room temperature. Subsequent to three final TBST rinses, immunoreactive bands were developed and imaged with a commercial enhanced chemiluminescence (ECL) (BL520A, Biosharp, Hefei, China) detection kit, and densitometric quantification of band grayscale was analyzed utilizing ImageJ software (version 1.54f) (NIH, Bethesda, MD, USA). Phosphorylated and total protein levels were detected on the same blots after membrane stripping with Western Stripping buffer (P0025, Shanghai, Beyotime).

### 2.6. Subcellular Protein Fractionation

Cytoplasmic and mitochondrial fractions were isolated using the Cytoplasm/Mitochondria Isolation Kit (C3601, Beyotime), strictly following the manufacturer’s protocol. Isolation purity was verified using β-actin as a cytoplasmic marker and TOM20 as a mitochondrial marker to ensure no cross-contamination. The isolated fractions were used for Western blot detection of TFAM expression.

### 2.7. Immunofluorescence Assay

Cell coverslips and frozen tissue sections were subjected to fixation in 4% paraformaldehyde for a 15-min duration. Membrane permeabilization was subsequently achieved via incubation in 0.5% Triton X-100 (ST1723, Beyotime, China) for 10 min at room temperature, prior to a 1-h blocking procedure using 5% BSA in a humidified atmosphere. Incubation steps were performed on sections using single primary antibodies or multiplex primary antibody combinations during overnight incubation at 4 °C: TOM20 (1:200, A27799, ABclonal)/PINK1 (1:200, A7131, ABclonal), TOM20 (1:200, A27799, ABclonal)/Parkin (1:200, A0968, ABclonal), TOM20 (1:200, A27799, ABclonal)/LC3 (1:200, A19665, ABclonal), TOM20 (1:200, AF1717, Beyotime)/LAMP1 (1:200, SC20011, Santa Cruz, Singapore), TOM20 (1:200, A27799, ABclonal)/TFAM (1:200, A23467, ABclonal), cleaved-caspase-3 (1:500, 68773-1-Ig, Proteintech), CD31 (1:800, 66065-2-Ig, Proteintech)/FLNC (1:200, A13018, ABclonal), CD31 (1:200, 28083-1-AP, Proteintech)/cleaved-caspase-3 (1:500, 68773-1-Ig, Proteintech), α-SMA(1:200, 67735-1-Ig, Proteintech)/FLNC(1:200, A13018, ABclonal). Following three PBS rinses, sections were treated with corresponding fluorophore-labeled secondary IgG antibodies (dilution ratio 1:200; AS037/AS039/AS073/AS054, ABclonal) and kept at room temperature for one hour, away from light. Following DAPI (C1006, Beyotime) counterstaining for 5 min to label cell nuclei, sections were mounted with an anti-fade fluorescent mounting medium. All fluorescence images were captured with an inverted fluorescence microscope (Leica DMI4000B, Wetzlar, Germany) and analyzed with ImageJ software.

Co-localization of TOM20 with PINK1 and TOM20 with Parkin was analyzed using Pearson’s colocalization coefficient. Co-localization of TOM20 with LC3 and TOM20 with LAMP1 was analyzed using Manders’ overlap coefficient. Representative images are shown for TOM20/TFAM, CD31/FLNC, and CD31/cleaved-caspase-3 co-localization, without statistical analysis.

### 2.8. ROS Detection

The levels of intracellular total ROS were detected with a DCFH-DA fluorescent probe kit (S0033S, Beyotime), and the levels of mitochondrial ROS were detected with a MitoSOX fluorescent probe kit (S0061S, Beyotime) following the manufacturer’s protocol. Fluorescence intensity of the acquired images was analyzed with ImageJ software.

### 2.9. TUNEL Assay

Apoptotic cell death was evaluated via Beyotime’s TUNEL detection kit (C1088, Beyotime) according to the manufacturer’s instructions. The percentage of TUNEL-positive cells was quantified using fluorescence microscopy.

### 2.10. Mitochondrial Membrane Potential Detection

Quantification of mitochondrial membrane potential was performed using a JC-1 Mitochondrial Membrane Potential Assay Kit (C2003S, Beyotime), with all operations carried out in strict accordance with the manufacturer’s operating guidelines. An inverted fluorescence microscope was applied to obtain all fluorescence micrographs. The relative mitochondrial membrane potential was determined by calculating the intensity ratio of red JC-1 aggregates to green JC-1 monomers, with all quantitative analyses processed through the ImageJ platform.

### 2.11. PicoGreen Fluorescence Assay for Cytosolic DNA Detection

Cytosolic free dsDNA was detected using PicoGreen dsDNA Quantitation Reagent (12641ES01, YENSEN, Shanghai, China). Briefly, after the indicated treatments, cells were fixed, permeabilized, and incubated with 1:400 diluted PicoGreen working solution for 30 min at room temperature in the dark. Fluorescence images were captured with an inverted fluorescence microscope under a FITC channel, with identical imaging parameters for all samples. Cytosolic PicoGreen fluorescence intensity was quantified using ImageJ: after background subtraction, the nucleus mask was generated based on the high-intensity nuclear PicoGreen signal, and the cytosolic region of interest (ROI) was obtained by subtracting the nucleus mask from the dilated whole-cell mask. The mean fluorescence intensity of each group was normalized to the control group. All experiments were repeated four times independently.

### 2.12. Transmission Electron Microscopy

Specimen pretreatment for TEM observation was conducted via the steps listed hereafter. For brief processing, cell pellets isolated from all experimental cohorts underwent primary fixation in 2.5% glutaraldehyde at 4 °C lasting two hours, prior to secondary fixation utilizing 1% osmium tetroxide with a 1-h incubation duration. Cellular sediments underwent progressive dehydration through ascending ethanol concentrations, after which epoxy resin embedding was performed. The embedded blocks were sliced into 70-nm ultra-thin specimens and subjected to dual heavy metal staining with uranyl acetate and lead citrate. Ultrastructural alterations of mitochondria, as well as the formation of autophagosomes and lysosomes, were observed and assessed with a transmission electron microscope.

### 2.13. Animal Experiments

Twelve 7-week-old male SPF-grade C57BL/6J wild-type mice and forty-eight 7-week-old male ApoE^−/−^ mice were purchased from Beijing Vital River Laboratory Animal Technology Co., Ltd. Mice were housed in an SPF-grade animal facility maintained at a constant temperature of 22 ± 1 °C, relative humidity of 50 ± 5%, and a 12-h light/dark cycle, with ad libitum access to standard diet and drinking water throughout the feeding period. After one week of adaptive feeding to acclimate to the housing conditions, the formal experimental interventions were implemented.

Forty-eight ApoE^−/−^ mice were subjected to random grouping and assigned to four separate experimental groups (n = 12 per group): ① High-fat-diet (HFD) group: Fed a high-fat diet (H10141, Beijing Huafukang Bioscience Co., Ltd., Beijing, China) for 12 weeks. ② HFD + normal saline (NS) group: Received 100 μL sterile normal saline via tail vein injection concurrently with the initiation of high-fat-diet feeding. ③ HFD + endothelial-specific FLNC overexpression (HFD+OE-FLNC) group: Received 100 μL Tie2 promoter-driven endothelial-specific AAV9-OE-FLNC (titer: 1 × 10^12^ vg/mL, VB240728-3264rfc, VB240728-3289rpf, VB240808-1120xny, VectorBuilder, Guangzhou, China) via tail vein injection concurrently with the initiation of high-fat-diet feeding. ④ HFD + scrambled overexpression negative control (HFD+OE-NC) group: Received 100 μL Tie2 promoter-driven endothelial-specific AAV9 expressing a non-functional scrambled sequence (titer: 1 × 10^12^ vg/mL, VectorBuilder) via tail vein injection concurrently with the initiation of high-fat-diet feeding. Twelve wild-type mice receiving a standard chow diet served as the normal control (C57) group.

Body weights were measured at 8, 14, and 20 weeks of age. After 12 weeks of feeding, mice were deeply anesthetized by intraperitoneal injection. The full-length aorta, bilateral carotid arteries, bilateral renal arteries, and bilateral iliac arteries were harvested from each mouse, and all tissues were labeled with individual mouse identification numbers. Five mice per group were randomly selected, and a small segment of the aortic arch and common carotid artery bifurcation (as indicated by red arrows in Figure S1A) was fixed in 4% paraformaldehyde (PFA, G1101, Servicebio, Wuhan, China) for frozen section preparation and subsequent histological staining. The remaining vascular tissues from these 5 mice, together with the entire vascular tissues from the other 7 mice per group, were used for isolation of primary endothelial cells and smooth muscle cells.

### 2.14. Isolation and Purity Verification of Mouse Primary Vascular Cells

Given the limited yield of primary aortic endothelial cells obtainable from a single mouse aorta, arterial vascular tissues from 12 mice per group were randomly divided into 4 subgroups, with 3 mice per subgroup. Primary aortic endothelial cells (PAECs) and primary aortic smooth muscle cells (PASMCs) were isolated using the collagenase perfusion method [26]. Vascular cells isolated from the 3 mice in each subgroup were pooled separately by cell type to generate one biological replicate, resulting in 4 independent biological replicates per experimental group. The purity of isolated primary cells was verified by Western blotting analysis of CD31 (endothelial cell identification marker) and α-smooth muscle actin (α-SMA, smooth muscle cell identification marker) expression.

### 2.15. Histological Staining

H&E staining: Aortic and carotid frozen sections (5 μm thick) were treated with routine HE staining procedures. Plaque morphology was observed under an optical microscope and photographed. Analysis of plaque area percentage was performed using ImageJ software.

Oil Red O staining assay: Following pre-rinsing with 60% isopropanol, the frozen sections were immersed in Oil Red O (C0157, Beyotime) working stain over a 30-min incubation period at room temperature under light-shielded conditions, differentiated with 60% isopropanol until the background was clear, and counterstained with hematoxylin. Lipid deposition was observed via a light microscope and photographed. ImageJ software was utilized to quantify the lipid area proportion of tissue sections.

### 2.16. Statistical Analysis

For TEM quantitative analysis, all experiments were performed with 4 independent biological replicates using cells from different passages. For each biological replicate, 5 non-overlapping fields with intact cell morphology were randomly selected for imaging and quantification, yielding a total of 20 valid observational fields per group. All counting procedures were conducted in a blinded manner by researchers unaware of group assignments to minimize subjective selection bias.

The percentage of damaged mitochondria was used as the core quantitative indicator to reflect the degree of mitochondrial ultrastructural injury. Mitochondria were classified as damaged if they met any of the following criteria: (i) obvious mitochondrial swelling; (ii) fractured, partially lost, or completely disappeared cristae; (iii) markedly decreased matrix electron density accompanied by vacuolization; (iv) rupture of the outer mitochondrial membrane. The percentage of damaged mitochondria per field was calculated as the ratio of the number of damaged mitochondria to the total number of mitochondria in the same field for intergroup comparison.

All quantitative data were first subjected to normality testing using the Shapiro–Wilk test and homoscedasticity testing using the Brown–Forsythe test. All datasets in this study were confirmed to follow a normal distribution with equal variance, justifying the use of parametric statistical tests. A minimum of four independent biological replicates were included for all experimental procedures. Quantitative data are expressed as mean ± standard error of the mean (mean ± SEM). Statistical analysis was performed with GraphPad Prism 9.0 software (GraphPad Software, San Diego, CA, USA). Comparisons between two groups were performed with paired Student’s *t*-test for paired clinical samples and unpaired Student’s *t*-test for independent groups. Comparisons among multiple groups were performed with one-way analysis of variance (ANOVA) followed by Tukey’s honestly significant difference (HSD) post hoc test. A *p*-value < 0.05 was considered statistically significant.

## 3. Results

### 3.1. FLNC Is Downregulated in Atherosclerotic Models, and Endothelial Cell-Specific Overexpression of FLNC Attenuates Atherosclerotic Plaque Progression in ApoE^−/−^ Mice

**Figure 1:**
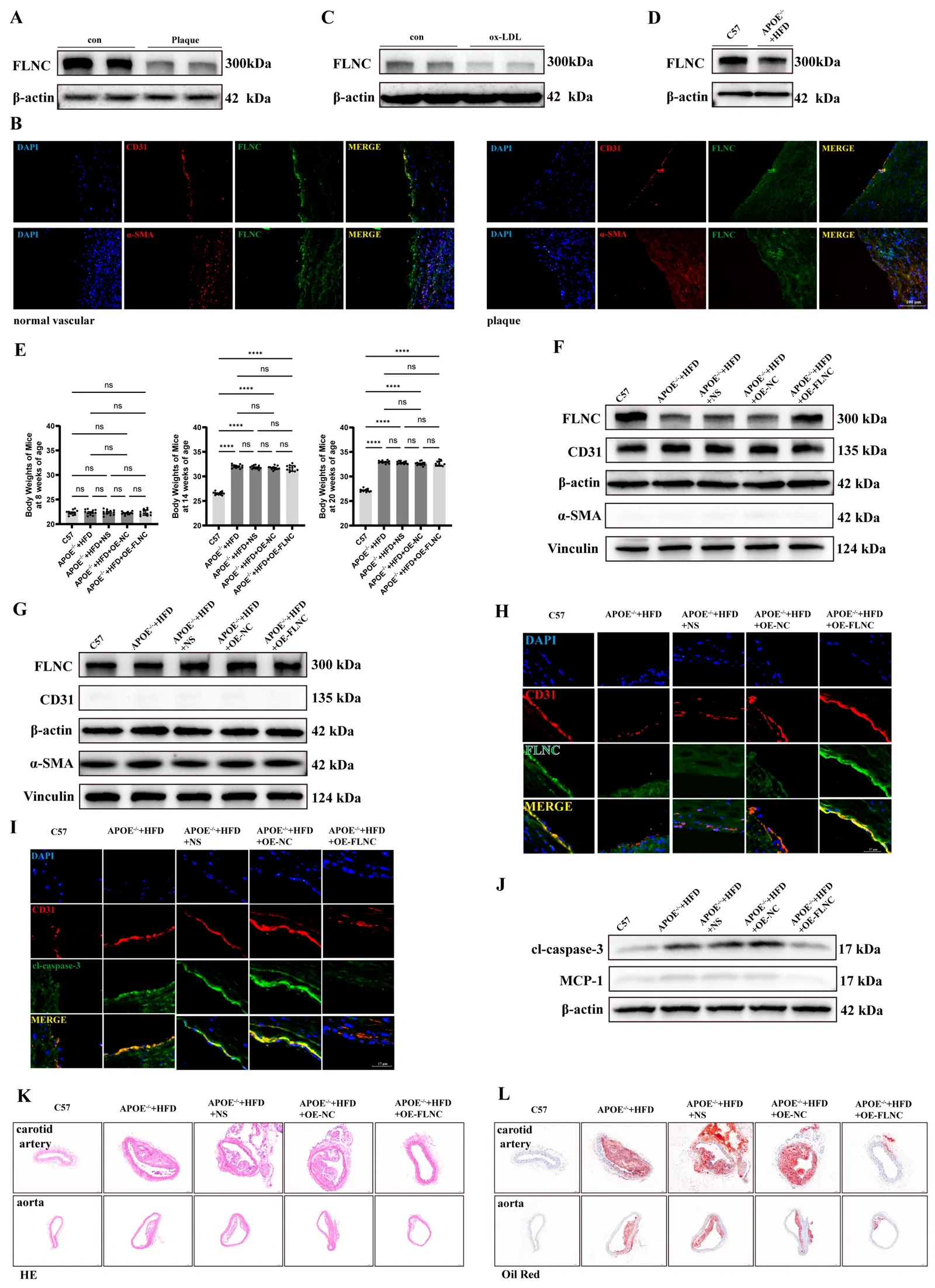
FLNC is downregulated in atherosclerotic models, and endothelial cell-specific overexpression of FLNC attenuates atherosclerotic plaque progression in ApoE^−/−^ mice. FLNC protein expression levels were detected in human clinical tissues, in vitro endothelial cell systems, and in vivo murine experimental models. We then validated the purity of isolated primary endothelial cells, confirmed the endothelial expression of FLNC in mouse aortas, and evaluated the functional effects of endothelial FLNC overexpression on endothelial apoptosis, inflammation and atherosclerotic plaque formation in ApoE^−/−^ mice. (**A**) Western blotting analysis of FLNC expression in human carotid atherosclerotic plaques and paired adjacent normal vascular tissues (n = 20). (**B**) Double immunofluorescence staining of FLNC (green) with CD31 (red, endothelial marker, upper) or α-SMA (red, smooth muscle marker, lower) in human carotid plaques and paired normal vessels, with DAPI (blue) nuclear counterstaining. Left: normal vessels; right: plaques; scale bar: 100 μm (n = 5). (**C**) Western blotting analysis of FLNC expression in HUVECs treated with 25 μg/mL ox-LDL for 24 h (n = 4). (**D**) Western blotting analysis of FLNC expression in primary aortic endothelial cells isolated from normal chow diet (NCD)-fed C57BL/6J mice and high-fat-diet (HFD)-fed ApoE^−/−^ mice (n = 4). (**E**) Weights were tracked for animals belonging to each experimental group at 8, 14, and 20 weeks of age (n = 12 per group). (**F**) Western blotting analysis of FLNC, CD31 and α-SMA (smooth muscle cell marker) expression in primary aortic endothelial cells (n = 4). No detectable α-SMA bands were observed, confirming high purity of isolated endothelial cells. (**G**) Western blotting analysis of FLNC, α-SMA and CD31 (endothelial cell marker) expression in primary aortic smooth muscle cells (n = 4). No detectable CD31 bands were observed, confirming high purity of isolated smooth muscle cells. (**H**) Co-immunofluorescence staining of CD31 (endothelial marker) and FLNC in mouse aortic sections (scale bar: 17 μm). (**I**) Co-immunofluorescence staining of CD31 and cleaved-caspase-3 in mouse aortic sections (scale bar: 17 μm). (**J**) Western blotting analysis of cleaved-caspase-3 and MCP-1 expression in primary aortic endothelial cells (n = 4). (**K**) Histological analysis of atherosclerotic plaques by H&E staining. Plaque areas in both carotid arteries and aortas were quantified; scale bar: 50 μm for carotid artery, 200 μm for aorta (n = 5). (**L**) Lipid content analysis by Oil Red O staining. Lipid contents in both carotid arteries and aortas were quantified; scale bar: 50 μm for carotid artery, 200 μm for aorta (n = 5). Data are presented as mean ± SEM. ns non-significant, **** *p* < 0.0001.

To explore the potential role of FLNC in atherosclerosis, we first examined FLNC expression levels in clinical carotid plaque samples, ox-LDL-induced endothelial injury models, and ApoE^−/−^ mouse atherosclerosis models. FLNC protein expression was significantly downregulated in carotid plaque tissues compared with paired adjacent normal vascular tissues from the same patients (Figure 1A and Figure S1B). Quantitative immunofluorescence analysis confirmed that FLNC expression in CD31-positive endothelial regions was significantly lower in atherosclerotic plaques than in paired adjacent normal vascular tissues, while FLNC expression in α-SMA-positive smooth muscle regions showed no significant difference between the two groups. These results indicate that FLNC downregulation in atherosclerotic lesions is predominantly restricted to the endothelial cell layer, supporting the endothelial-specific regulatory role of FLNC in atherosclerosis (Figure 1B and Figure S1C). Compared with normal untreated HUVECs and HAECs, cells exposed to 25 μg/mL ox-LDL for 24 h exhibited significant reductions in FLNC protein abundance in vitro (Figure 1C and Figure S1D,E). In vivo, we further confirmed that FLNC protein expression was significantly reduced in primary aortic endothelial cells isolated from HFD-fed ApoE^−/−^ mice compared with normal chow diet (NCD)-fed C57BL/6J wild-type mice (Figure 1D and Figure S1F). These findings indicate that FLNC expression is markedly downregulated during the initiation and progression of atherosclerosis and may be implicated in the pathological process of vascular endothelial injury.

To validate the in vivo protective effect of FLNC against atherosclerosis, we generated ApoE^−/−^ mice with endothelial cell-specific FLNC overexpression driven by the Tie2 promoter. Measurements taken at 8 weeks of age indicated that animal body weights were comparable among all experimental cohorts. At 14 and 20 weeks of age, C57BL/6J wild-type mice exhibited significantly lower body weights than the other four groups, with no statistically significant differences within these cohorts (Figure 1E). Western blotting analysis of primary aortic endothelial cells revealed that no detectable bands for the smooth muscle cell marker α-SMA were observed in any group, confirming the high purity of the isolated endothelial cells. Notably, FLNC protein levels were comparable among the ApoE^−/−^+HFD, ApoE^−/−^+HFD+NS, and ApoE^−/−^+HFD+OE-NC groups. Consistent with our earlier findings, FLNC expression was significantly downregulated in aortic endothelial cells from ApoE^−/−^ mice under high-fat feeding compared with normal chow-raised C57BL/6J wild-type mice. In contrast, endothelial-specific overexpression of FLNC resulted in a significant upregulation of FLNC protein levels in the ApoE^−/−^+HFD+OE-FLNC group compared with the ApoE^−/−^+HFD group, confirming successful transduction and overexpression (Figure 1F and Figure S1G). Western blotting analysis of primary aortic smooth muscle cells showed no detectable bands for CD31, a specific marker of endothelial cells, in any group, confirming the high purity of the isolated smooth muscle cells. Furthermore, FLNC protein levels were similar across all groups, demonstrating the excellent endothelial specificity of the AAV9-Tie2 vector with no off-target expression in smooth muscle cells (Figure 1G and Figure S1H). Co-immunofluorescence staining demonstrated that co-localization of CD31 (an endothelial marker) and FLNC was visibly stronger in the aortic sections of C57BL/6J wild-type mice and ApoE^−/−^+HFD+OE-FLNC mice compared with the other three groups (Figure 1H). Similarly, co-localization of CD31 and cleaved-caspase-3 was markedly reduced in the aortas of C57BL/6J wild-type mice and ApoE^−/−^+HFD+OE-FLNC mice relative to the other three groups (Figure 1I). Western blotting analysis of primary aortic endothelial cells further confirmed that the protein abundances of cleaved-caspase-3 and MCP-1 were comparable among the ApoE^−/−^+HFD, ApoE^−/−^+HFD+NS, and ApoE^−/−^+HFD+OE-NC groups. Compared with the ApoE^−/−^+HFD group, endothelial-specific FLNC overexpression significantly attenuated the high-fat-diet-induced upregulation of these pro-apoptotic and pro-inflammatory mediators (Figure 1J and Figure S1I).

Histological analysis revealed that the aortic intima of NCD-fed C57BL/6J wild-type mice was smooth with no evident atherosclerotic plaque formation. Extensive plaque deposition was observed in the aortas of the ApoE^−/−^+HFD, ApoE^−/−^+HFD+NS, and ApoE^−/−^+HFD+OE-NC groups, with no statistically significant differences in plaque area or lipid content among these three groups. In contrast, endothelial-specific FLNC overexpression significantly reduced both aortic plaque area and lipid content compared with the ApoE^−/−^+HFD group (Figure 1K,L and Figure S1J,K). Collectively, these results demonstrated that endothelial-specific overexpression of FLNC significantly inhibited aortic endothelial cell apoptosis and inflammation, reduced atherosclerotic plaque formation, and thereby attenuated the progression of atherosclerosis in ApoE^−/−^ mice.

### 3.2. FLNC Activates PINK1/Parkin-Mediated Mitophagy

**Figure 2:**
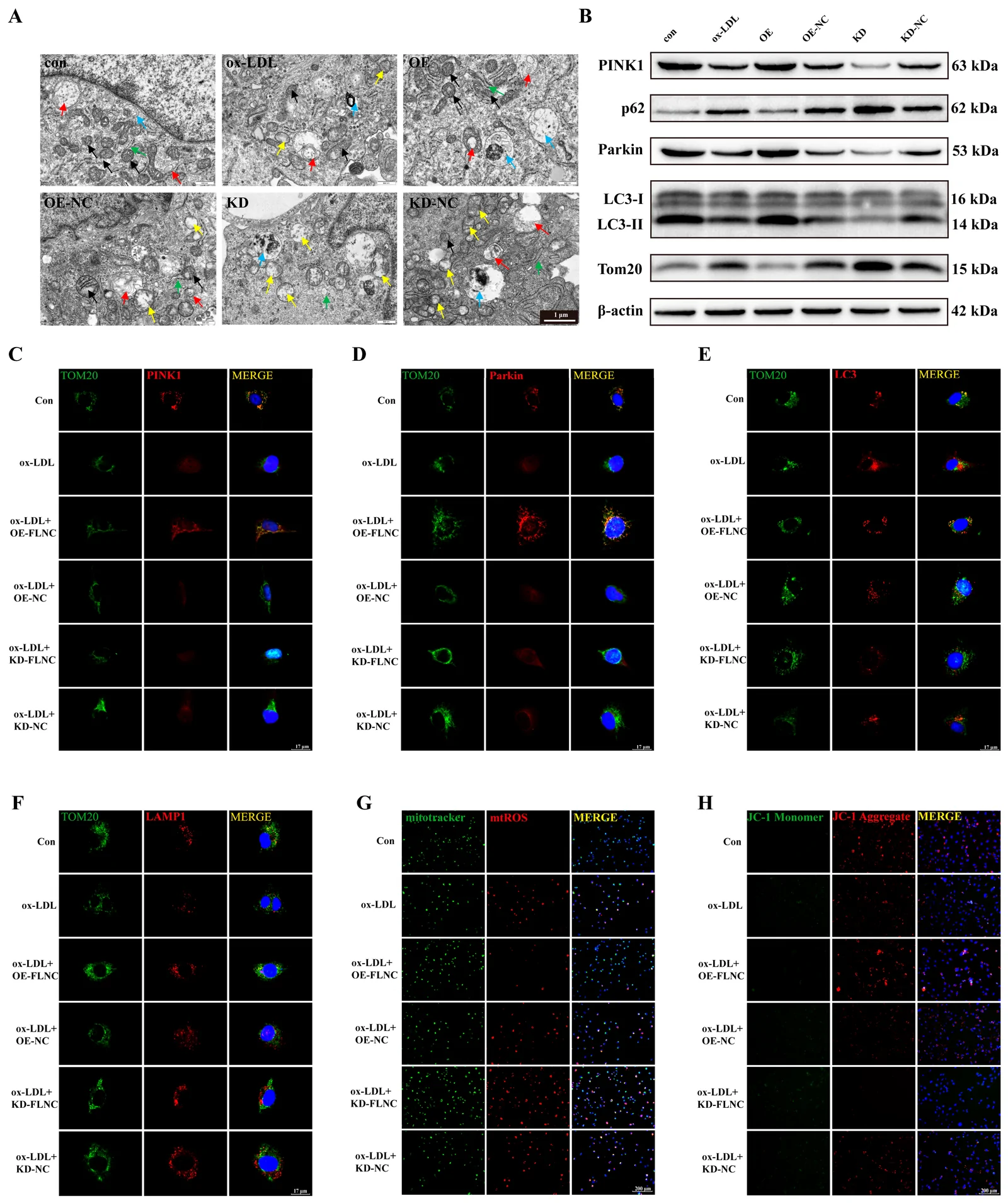
FLNC activates PINK1/Parkin-mediated mitophagy. Stable HUVEC lines with FLNC overexpression (OE) or knockdown (KD) were established using CRISPRa and CRISPRi systems, respectively, and the effect of FLNC on PINK1/Parkin-mediated mitophagy was evaluated. (**A**) Transmission electron microscopy (TEM) analysis of mitochondrial ultrastructure and autophagic flux. Representative images are shown. Black arrows: normal mitochondria; green arrows: endoplasmic reticulum; red arrows: autophagosomes; blue arrows: autolysosomes; yellow arrows: damaged mitochondria (scale bar: 1 μm). (**B**) Western blotting analysis of mitophagy-related proteins (PINK1, Parkin, LC3II, p62, and TOM20) (n = 4). (**C**,**D**) Co-immunofluorescence staining of TOM20 with PINK1 or Parkin (scale bar: 17 μm, n = 4). (**E**,**F**) Co-immunofluorescence staining of TOM20 with LC3 (autophagosome marker) or LAMP1 (lysosome marker) (scale bar: 17 μm, n = 4). (**G**) Mitochondrial ROS (mtROS) levels were measured by MitoSOX staining (scale bar: 200 μm, n = 4). (**H**) JC-1 fluorescence labeling was applied to assess mitochondrial membrane potential (scale bar: 200 μm, n = 4).

For the purpose of exploring how FLNC exerts protective functions against endothelial injury at the molecular level, we first generated stable HUVEC cell lines and HAEC cell lines with endogenous FLNC activation (CRISPRa) and inhibition (CRISPRi). In HUVECs, Western blotting confirmed that FLNC protein levels were significantly elevated in the overexpression (OE) group compared with the OE-negative control (OE-NC) group and significantly reduced in the knockdown (KD) group compared with the KD-negative control (KD-NC) group. Immunofluorescence staining further validated the transfection efficiency (Figure S2A–C). The same validation in HAECs confirmed successful FLNC overexpression and knockdown (Figure S3A–C). In HUVECs, TEM was performed to examine mitochondrial ultrastructure and autophagic flux. In control cells, the cytoplasmic architecture was well-preserved, with mitochondria displaying regular morphology, uniform size, intact membranes, well-defined cristae, and a dense matrix (black arrows). The endoplasmic reticulum (ER) was well-organized without dilation (green arrows), and abundant autophagosomes (red arrows) and autolysosomes (blue arrows) were observed. Relative to untreated control HUVECs, cells subjected to ox-LDL stimulation exhibited mild cellular injury, with partial mitochondrial cristae fragmentation and loss, and decreased matrix density (yellow arrows). The ER structure remained intact, while the number of autophagosomes and autolysosomes decreased. FLNC-overexpressing cells showed a cellular morphology nearly identical to that of control cells. Cells in the OE-NC and KD-NC groups exhibited changes similar to those in the ox-LDL-treated group. FLNC-knockdown cells showed severe cellular damage, with widespread mitochondrial destruction, complete cristae loss, and vacuolar degeneration. ER dilation was also evident, and the number of autophagosomes and autolysosomes was markedly reduced (Figure 2A). Quantitative analysis of the percentage of damaged mitochondria further confirmed these morphological observations: the proportion of damaged mitochondria was significantly elevated in the ox-LDL group compared with the control group; FLNC overexpression significantly reduced the percentage of damaged mitochondria relative to the ox-LDL group, whereas FLNC knockdown further increased it, with no significant differences observed among the ox-LDL, OE-NC, and KD-NC groups (Figure S2D). Furthermore, in HUVECs, we examined the activation level of the PINK1/Parkin-mediated mitophagy pathway and mitochondrial homeostasis-related indicators. Notably, all quantitative indices showed no significant differences between the ox-LDL, OE-NC, and KD-NC groups. Western blotting revealed that compared with untreated control cells, ox-LDL treatment significantly decreased the protein levels of PINK1, Parkin, and LC3II, while increasing the levels of p62 and TOM20, indicating impaired mitophagy in ox-LDL-injured endothelial cells. FLNC overexpression significantly reversed these protein expression changes, whereas FLNC knockdown further exacerbated mitophagy inhibition (Figure 2B and Figure S2E). Consistent with the findings in HUVECs, parallel expression patterns of these mitophagy-related proteins were observed in HAECs (Figure S3D). In HUVECs, co-immunofluorescence staining analysis demonstrated that ox-LDL stimulation significantly reduced the co-localization levels of TOM20 with PINK1 and TOM20 with Parkin. FLNC overexpression significantly restored this co-localization, whereas FLNC knockdown further reduced it (Figure 2C,D and Figure S2F,G). Parallel co-localization patterns were observed in HAECs (Figure S3E,F). Similarly, co-localization analysis of TOM20 with LC3 (an autophagosome marker) and LAMP1 (lysosome marker) revealed that ox-LDL stimulation inhibited mitochondrial fusion with autophagosomes and lysosomes. FLNC overexpression markedly promoted this process, whereas FLNC knockdown further suppressed it (Figure 2E,F and Figure S2H,I). Parallel co-localization patterns were observed in HAECs (Figure S3G,H). In HUVECs, the assessment of mitochondrial function demonstrated that ox-LDL stimulation significantly increased mitochondrial ROS (mtROS) levels and decreased mitochondrial membrane potential. FLNC overexpression markedly inhibited mtROS accumulation and preserved mitochondrial membrane potential, whereas FLNC knockdown further augmented mtROS accumulation and aggravated mitochondrial membrane depolarization (Figure 2G,H and Figure S2I,J). These findings demonstrated that FLNC activates the signaling axis of PINK1 and Parkin, which drives the progression of mitophagy, thereby preserving mitochondrial structure and function.

### 3.3. FLNC Inhibits Apoptosis and Oxidative Stress, Suppresses mtDNA Release and Blocks the cGAS-STING Inflammatory Pathway

To verify FLNC’s protective effect on endothelial cells, we examined the levels of apoptosis and oxidative stress in each group of HUVECs. Notably, all quantitative experimental readouts showed no statistically distinct variations among the ox-LDL, OE-NC, and KD-NC groups. Analysis of apoptosis-related proteins revealed that cleaved-caspase-3 abundances rose markedly in cells exposed to ox-LDL relative to vehicle-treated controls; FLNC overexpression markedly attenuated this increase, whereas FLNC knockdown further augmented cleaved-caspase-3 expression (Figure S4A). Immunofluorescence detection of cleaved-caspase-3 showed consistent trends with Western blotting results (Figure S4B). TUNEL staining confirmed that ox-LDL exposure robustly elevated endothelial cell apoptosis, an effect that was markedly reversed by FLNC overexpression and further exacerbated by FLNC knockdown (Figure S4C). Measurement of intracellular reactive oxygen species (ROS) revealed that ox-LDL treatment significantly elevated ROS levels compared with untreated control cells; FLNC overexpression markedly inhibited ox-LDL-induced ROS accumulation, whereas FLNC knockdown further augmented ROS accumulation (Figure S4D). All the above apoptosis and oxidative stress phenotypes exhibited parallel patterns in HAECs (Figure S5A–D). Consistent with the above observations, FLNC confers a potent protective activity resisting damage provoked by ox-LDL, which relieves apoptosis and oxidative stress injury in HUVECs and HAECs.

**Figure 3:**
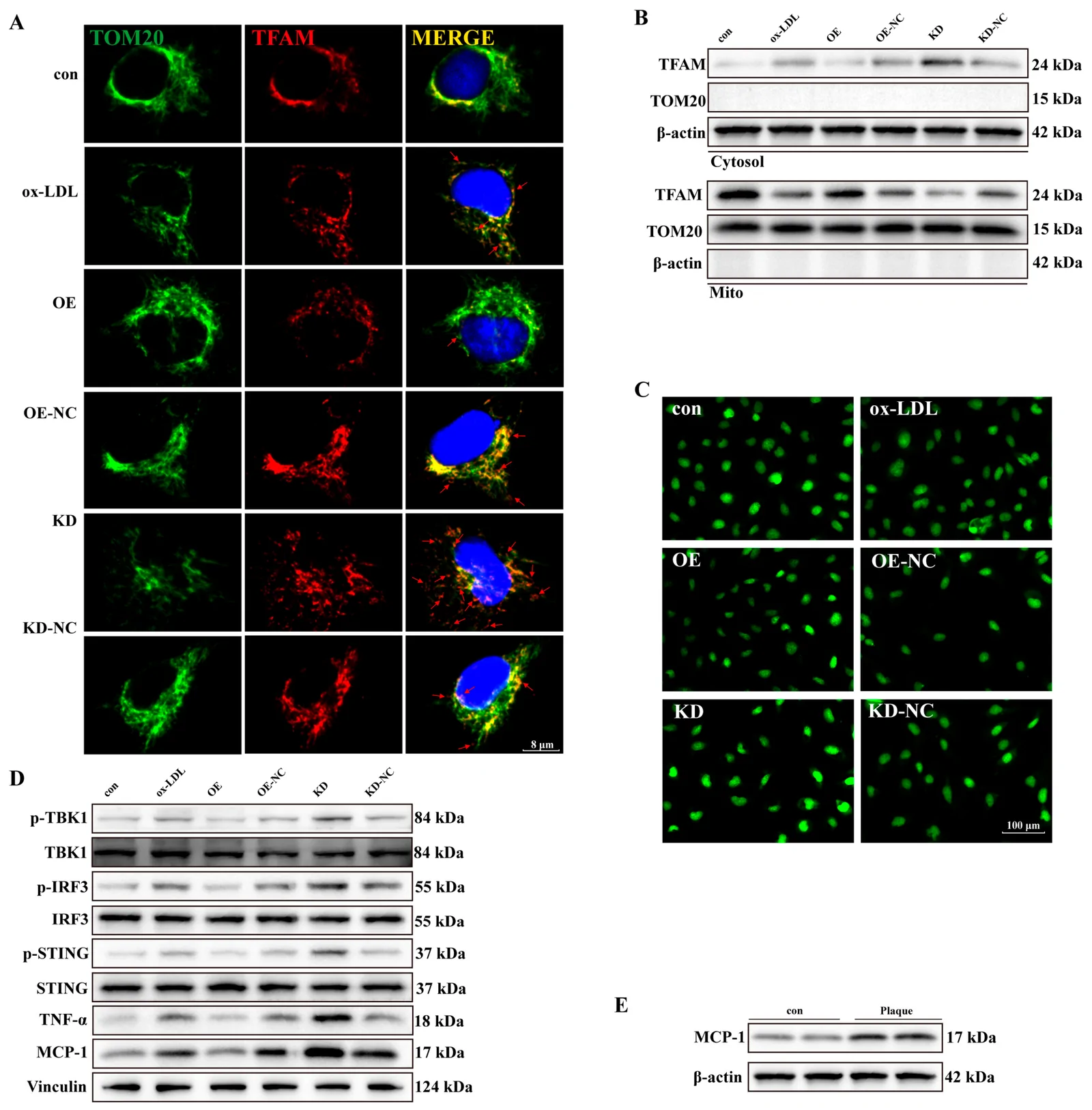
FLNC suppresses mtDNA release and inhibits the cGAS-STING inflammatory pathway. The effect of FLNC on mitochondrial DNA leakage and cGAS-STING signaling cascade induction was investigated. (**A**) Co-immunofluorescence staining of TOM20 (mitochondrial marker, green) and TFAM (mtDNA-binding protein, red). Red arrows indicate TFAM-positive regions not co-localized with TOM20 (scale bar: 8 μm). (**B**) Subcellular fractionation followed by Western blotting was applied for the measurement of TFAM levels across cytoplasmic and mitochondrial compartments (n = 4). The purity of the isolated fractions was confirmed by the absence of TOM20 in cytoplasmic extracts and β-actin in mitochondrial preparations. (**C**) PicoGreen fluorescence staining of cytosolic double-stranded DNA in HUVECs (n = 4) (scale bar: 100 μm). (**D**) Western blotting to profile key mediators of cGAS-STING inflammatory axis components and downstream pro-inflammatory cytokines (n = 4). (**E**) Western blotting quantifying MCP-1 protein levels within human carotid plaques and corresponding non-lesioned perilesional vasculature (n = 20). Phosphorylated and total protein levels were detected on the same blots after membrane stripping with Western Stripping buffer.

To investigate the downstream effects of FLNC-mediated mitophagy activation in HUVECS, we assessed mtDNA release and cGAS-STING inflammatory pathway activity. Co-immunofluorescence staining for TOM20 (a mitochondrial outer membrane marker, green) and TFAM (an mtDNA-binding protein, red) revealed co-localization of the two signals in control and FLNC-overexpressing (OE) cells. In contrast, partial dissociation of TFAM from TOM20 was observed in ox-LDL-treated, OE-NC, and KD-NC cells, with scattered TFAM signals detected in the cytoplasm. Notably, extensive cytoplasmic TFAM release was evident in FLNC-knockdown (KD) cells (Figure 3A). Subcellular fractionation followed by Western blotting confirmed the purity of the isolated fractions: no TOM20 was detected in cytoplasmic extracts, and no β-actin was detected in mitochondrial preparations. Consistent with our previous findings, no significant differences in cytoplasmic TFAM levels were observed between ox-LDL-treated, OE-NC, and KD-NC cells. Ox-LDL stimulation significantly increased cytoplasmic TFAM accumulation compared with untreated controls; this effect was markedly attenuated by FLNC overexpression and further augmented by FLNC knockdown. Mitochondrial TFAM levels exhibited an inverse pattern, mirroring the changes observed in the cytoplasm (Figure 3B and Figure S4E). To further directly validate the level of cytosolic mtDNA release, we used PicoGreen fluorescent dye to specifically label cytosolic double-stranded DNA, and quantified the cytosolic fluorescence intensity with ImageJ software [27]. The results showed that ox-LDL treatment significantly elevated cytosolic double-stranded DNA levels compared with the control group. FLNC overexpression markedly inhibited ox-LDL-induced cytosolic DNA accumulation, whereas FLNC knockdown further exacerbated cytosolic DNA release. No significant differences were observed among the ox-LDL, OE-NC, and KD-NC groups. Parallel cytosolic DNA accumulation patterns were observed in HAECs (Figure S5E). This result was fully consistent with the trend of cytoplasmic TFAM levels detected by subcellular fractionation, providing direct quantitative evidence for FLNC-regulated mtDNA release (Figure 3C and Figure S4F). Western blotting analysis of cGAS-STING pathway components and downstream inflammatory mediators revealed no significant differences between ox-LDL-treated, OE-NC, and KD-NC cells. Ox-LDL exposure markedly triggered the cGAS-STING cascade, reflected by enhanced phosphorylation of STING, TBK1, and IRF3, along with elevated expression of the pro-inflammatory cytokines TNF-α and MCP-1. FLNC overexpression significantly suppressed this pathway activation, whereas FLNC knockdown further exacerbated the inflammatory response (Figure 3D and Figure S4G). Parallel expression and phosphorylation patterns of these cGAS-STING pathway components were observed in HAECs (Figure S5F). We further detected total eNOS and phosphorylated eNOS (p-eNOS, Ser1177) levels. Total eNOS expression was comparable across all groups, while p-eNOS showed an opposite trend to inflammatory proteins such as p-STING: ox-LDL reduced p-eNOS level, FLNC overexpression reversed this decrease, and FLNC knockdown further aggravated p-eNOS downregulation, with no significant difference among ox-LDL, OE-NC and KD-NC groups (Figure S4H). Parallel expression patterns of total eNOS and p-eNOS were observed in HAECs (Figure S5G). This result provides direct functional evidence for FLNC-mediated endothelial protection. Consistent with our in vitro findings, analysis of human clinical samples demonstrated that MCP-1 protein levels were significantly higher in carotid atherosclerotic plaques than in paired adjacent normal vascular tissues (Figure 3E and Figure S4I). These results indicated that FLNC inhibited mtDNA release by activating mitophagy, thereby inhibiting the cGAS-STING-mediated inflammatory signaling axis and suppressing inflammation-related cytokine release.

### 3.4. The Protective Effect of FLNC Depends on Mitophagy

**Figure 4:**
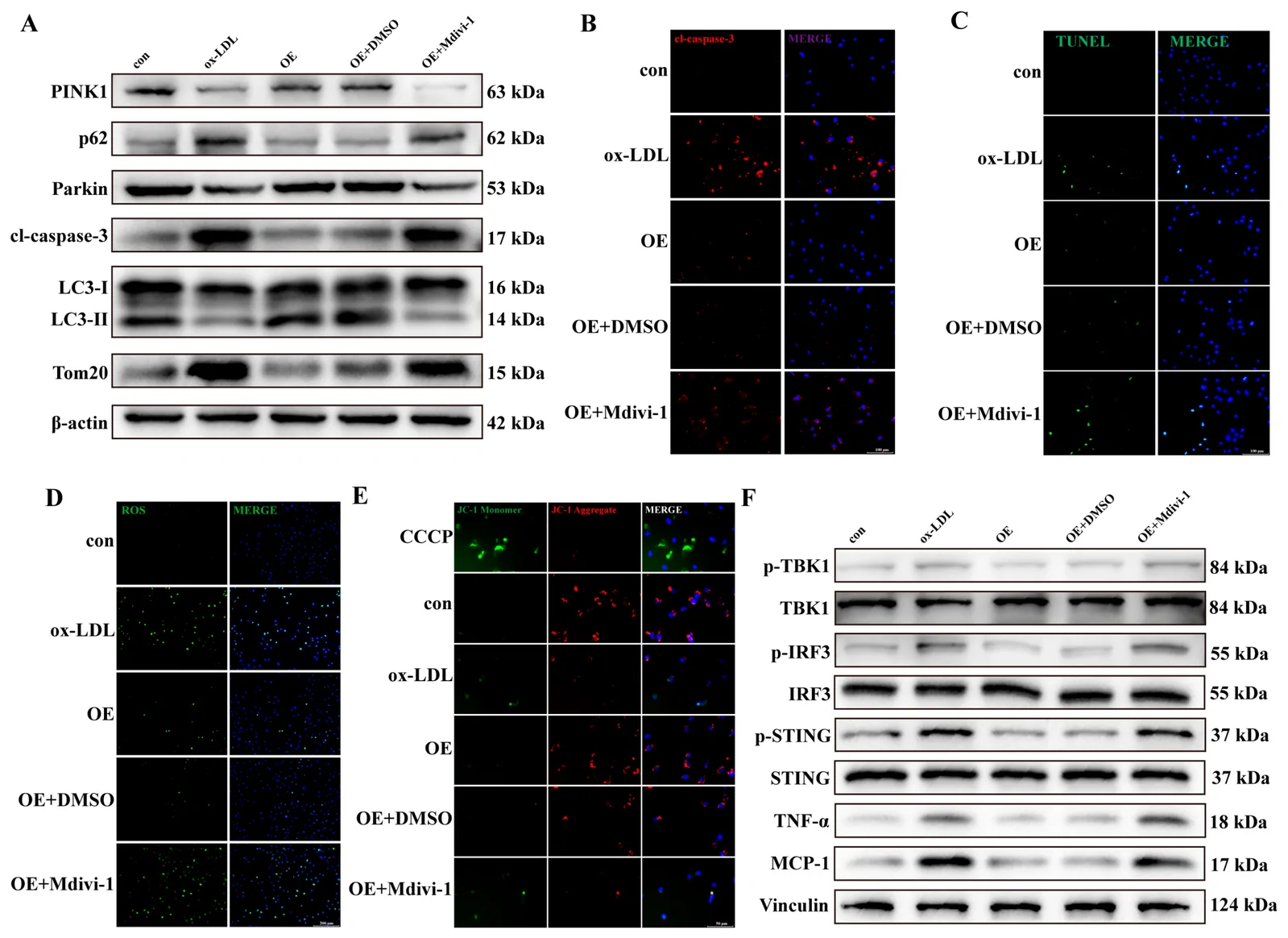
FLNC exerts its protective effects in a mitophagy-dependent manner. Mdivi-1, a pharmacologic blocker of mitophagy, was utilized for rescue experiments. (**A**) Western blotting analysis of mitophagy-related proteins and cleaved-caspase-3. PINK1 and Parkin (n = 4); LC3II, p62, TOM20, and cleaved-caspase-3 (n = 8). (**B**) Immunofluorescent labeling of cell coverslips targeting cleaved caspase-3 (scale bar: 100 μm, n = 4). (**C**) TUNEL assay was used to detect endothelial cell apoptosis (scale bar: 100 μm, n = 4). (**D**) Intracellular ROS levels were measured by DCFH-DA staining (scale bar: 200 μm, n = 4). (**E**) JC-1 fluorochrome was utilized for the quantification of mitochondrial membrane potential (scale bar: 100 μm, n = 4). (**F**) Western blotting analysis of cGAS-STING inflammatory signaling axis components and downstream pro-inflammatory cytokines (n = 4). Phosphorylated and total protein levels were detected on the same blots after membrane stripping with Western Stripping buffer.

To verify the necessity of mitophagy in the endothelial protective effects of FLNC, we performed rescue experiments using Mdivi-1, a widely used pharmacological inhibitor of mitochondrial fission and mitophagy. Consistent with earlier experimental data, ox-LDL treatment induced significant endothelial injury, as evidenced by impaired mitophagy, increased apoptosis, and excessive oxidative stress. None of the measured quantitative indices exhibited significant differences between the OE group and the OE+DMSO vehicle control group. Western blotting demonstrated that relative to the ox-LDL group, FLNC overexpression markedly elevated the protein levels of PINK1, Parkin, and LC3II, while downregulating the expression of p62, TOM20, and cleaved-caspase-3; notably, Mdivi-1 treatment completely abrogated the mitophagy activation and anti-apoptotic effects of FLNC (Figure 4A and Figure S6A). Immunofluorescence staining for cleaved-caspase-3 yielded consistent results (Figure 4B and Figure S6B). TUNEL apoptosis assay demonstrated that FLNC overexpression significantly reduced the ox-LDL-induced endothelial cell apoptosis rate, which was completely reversed by Mdivi-1 treatment (Figure 4C and Figure S6C). Assessment of oxidative stress and mitochondrial function showed that Mdivi-1 treatment also abolished the inhibitory effect of FLNC on ROS accumulation and its protective effect on mitochondrial membrane potential (Figure 4D,E and Figure S6D,E). Furthermore, Western blotting analysis showed that FLNC overexpression markedly downregulated the phosphorylation levels of STING, TBK1, and IRF3, alongside the expression of the downstream pro-inflammatory mediators MCP-1 and TNF-α, compared with the ox-LDL group. Mdivi-1 treatment completely reversed the inhibitory effect of FLNC on the cGAS-STING inflammatory pathway, resulting in significant upregulation of phosphorylated STING, TBK1, IRF3, and downstream inflammatory mediators (Figure 4F and Figure S6F). Collectively, these results illustrated that mitophagy suppression was sufficient to abolish all vascular endothelial protective benefits derived from FLNC upregulation, confirming that the protective effects of FLNC depend on the activation of mitophagy.

### 3.5. The Protective Effect of FLNC Requires the PINK1/Parkin Signaling Pathway

**Figure 5:**
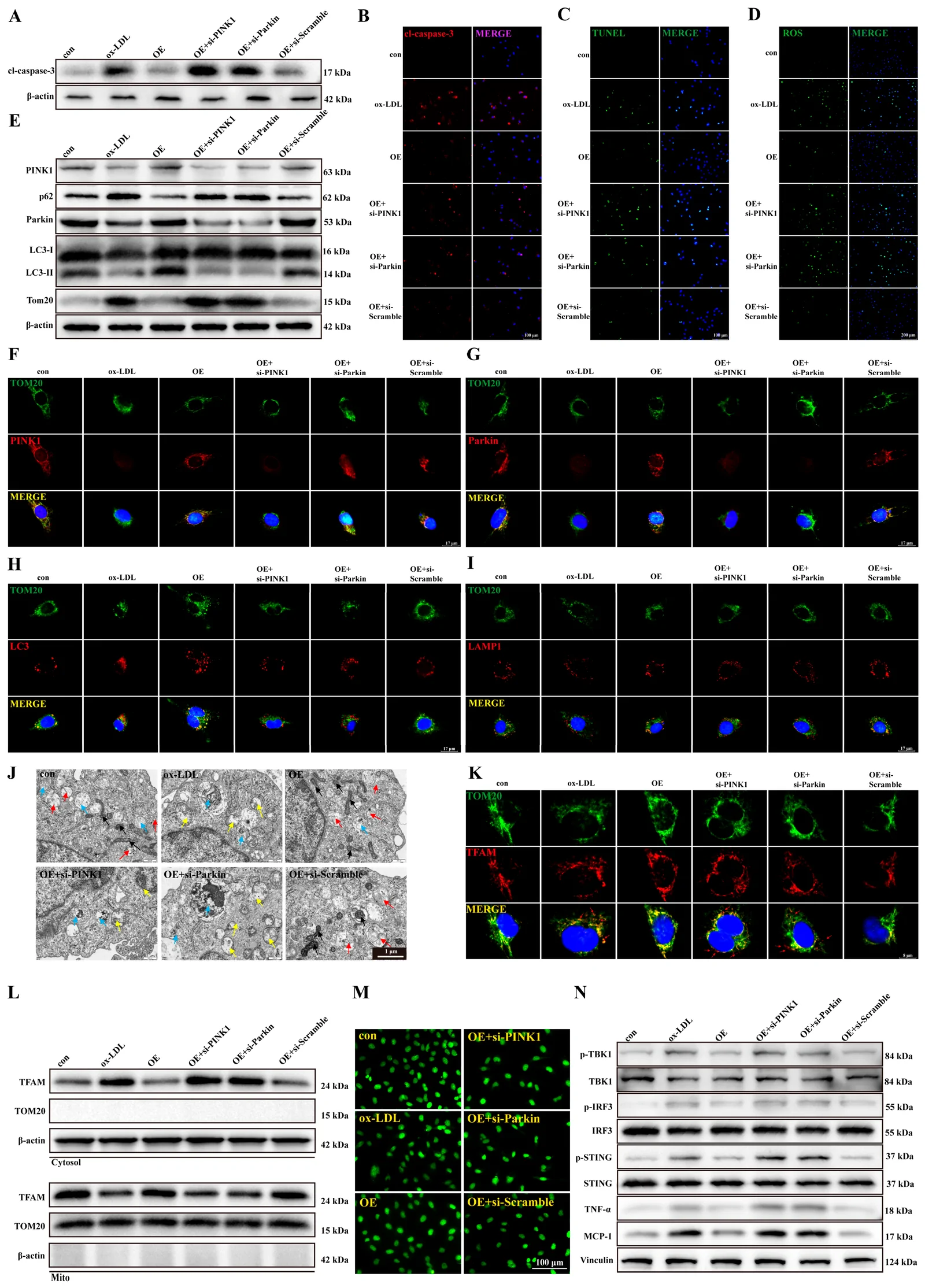
FLNC exerts its protective effects via the PINK1/Parkin signaling pathway. Rescue experiments were performed by transfecting cells with siRNAs targeting PINK1 or Parkin. (**A**) Western blotting analysis of cleaved-caspase-3 expression (n = 4). (**B**) Immunofluorescence staining targeting cleaved-caspase-3 (scale bar: 100 μm, n = 4). (**C**) TUNEL assay was used to detect endothelial cell apoptosis (scale bar: 100 μm, n = 4). (**D**) DCFH-DA staining was applied to quantify intracellular reactive oxygen species (scale bar: 200 μm, n = 4). (**E**) Western blotting analysis of mitophagy-related proteins (n = 4). (**F**,**G**) Dual immunofluorescence staining was performed for TOM20 paired with PINK1, and TOM20 paired with Parkin separately; co-localization was quantified using Pearson correlation coefficient (scale bar: 17 μm, n = 4). (**H**,**I**) Co-immunofluorescence staining of TOM20 with LC3 (autophagosome marker) and LAMP1 (lysosome marker); co-localization was quantified using Manders overlap coefficient (scale bar: 17 μm, n = 4). (**J**) TEM analysis of mitochondrial ultrastructure and autophagic flux. Black arrows: normal mitochondria; red arrows: autophagosomes; blue arrows: autolysosomes; yellow arrows: damaged mitochondria (scale bar: 1 μm). (**K**) Co-immunofluorescence staining of TOM20 and TFAM (scale bar: 8 μm). (**L**) Subcellular fractionation followed by Western blotting was performed to detect cytoplasmic TFAM levels (n = 4). The purity of the isolated fractions was confirmed by the absence of TOM20 in cytoplasmic extracts and β-actin in mitochondrial preparations. (**M**) PicoGreen fluorescence staining of cytosolic double-stranded DNA in HUVECs (n = 4) (scale bar: 100 μm). (**N**) Protein levels of cGAS-STING axis members as well as downstream pro-inflammatory mediators were determined via Western blotting (n = 4). Phosphorylated and total protein levels were detected on the same blots after membrane stripping with Western Stripping buffer.

To further verify whether activation of the PINK1/Parkin signaling axis is required for FLNC-conferred vascular endothelial protective effects, we performed rescue experiments using siRNA-mediated silencing of PINK1 or Parkin in HUVECs and HAECs. Western blotting revealed that transfection with si-PINK1 or si-Parkin resulted in significant downregulation of the respective target proteins in HUVECs, confirming successful silencing efficiency (Figure S7A,B). Parallel silencing efficiency of si-PINK1 and si-Parkin was confirmed in HAECs (Figure S8A,B). All quantified readouts exhibited no statistically distinguishable discrepancies between the OE group and the OE+si-Scramble negative control group. Western blotting revealed that compared with the ox-LDL group, FLNC overexpression significantly reduced cleaved-caspase-3 levels in HUVECs; whereas genetic knockdown of PINK1 or Parkin completely abrogated the anti-apoptotic effect of FLNC (Figure 5A and Figure S7C). Parallel expression patterns of cleaved-caspase-3 were confirmed in HAECs by Western blotting (Figure S8C). Immunofluorescence staining for cleaved-caspase-3 yielded consistent results of HUVECs (Figure 5B and Figure S7D). TUNEL assay and assessment of intracellular ROS further confirmed that knockdown of PINK1 or Parkin significantly reversed FLNC’s defensive capacity against HUVECs apoptosis and oxidative injury (Figure 5C,D and Figure S7E,F).

Western blot detection of mitophagy marker proteins demonstrated that genetic depletion of PINK1 or Parkin entirely eliminated the stimulatory effect of FLNC on the PINK1/Parkin axis in HUVECs, significantly reducing LC3-II expression levels and increasing p62 and TOM20 expression levels (Figure 5E and Figure S7G). Parallel expression patterns of these mitophagy marker proteins were confirmed in HAECs (Figure S8C). Co-immunofluorescence staining analysis demonstrated that in HUVECs the co-localization of TOM20 with PINK1, Parkin, LC3, and LAMP1 was markedly elevated within the OE group relative to the ox-LDL group; knockdown of PINK1 significantly reduced all these co-localization events, while knockdown of Parkin significantly reduced the co-localization of TOM20 with Parkin, LC3, and LAMP1, thereby reversing FLNC-induced mitophagy (Figure 5F–I and Figure S7H–K). In HUVECs, TEM was performed to examine mitochondrial ultrastructure and autophagic flux. In control cells, the cytoplasmic architecture was well-preserved, with mitochondria displaying normal morphology, intact membranes, well-defined cristae, and a dense matrix (black arrows). Abundant autophagosomes (red arrows) and autolysosomes (blue arrows) were also observed. Mitochondrial morphology in FLNC-overexpressing cells and OE+si-Scramble cells was nearly identical to that in control cells, with abundant autophagic structures. In contrast, mitochondria in ox-LDL-treated, OE+si-PINK1, and OE+si-Parkin cells exhibited extensive damage, including partial membrane rupture, cristae fragmentation, reduced matrix density, and vacuolization (yellow arrows), coupled with a prominent decrease in the number of autophagosomes and autolysosomes (Figure 5J). Quantitative analysis of the percentage of damaged mitochondria further corroborated the above morphological observations. ox-LDL stimulation significantly elevated the proportion of damaged mitochondria compared with the control group, while FLNC overexpression markedly reduced the percentage of damaged mitochondria to a level comparable to that of the control group. No significant difference was observed between the OE+si-Scramble negative control group and the OE group. In contrast, knockdown of PINK1 or Parkin completely abrogated the mitochondrial protective effect of FLNC, with the proportion of damaged mitochondria returning to a level comparable to the ox-LDL group and significantly higher than that in the OE group (Figure S7L).

In HUVECs, co-immunofluorescence staining for TOM20 and TFAM revealed co-localization of the two signals in control, FLNC-overexpressing, and OE+si-Scramble cells. In contrast, abundant cytoplasmic free TFAM signals were observed in ox-LDL-treated, OE+si-PINK1, and OE+si-Parkin cells (Figure 5K). Subcellular fractionation followed by Western blotting further confirmed that knockdown of PINK1 or Parkin significantly reversed FLNC-mediated inhibition of mtDNA release into the cytoplasm (Figure 5L and Figure S7M). PicoGreen fluorescence quantification yielded consistent results, confirming that knockdown of PINK1 or Parkin reversed FLNC-mediated inhibition of cytosolic mtDNA accumulation (Figure 5M and Figure S7N). Parallel PicoGreen fluorescence patterns were confirmed in HAECs (Figure S8D). Furthermore, knockdown of PINK1 or Parkin also completely abrogated the inhibitory effect of FLNC on the cGAS-STING inflammatory pathway in HUVECs (Figure 5N and Figure S7O). Identical inflammatory pathway regulatory patterns were observed in HAECs (Figure S8E). Collectively, these findings demonstrated that genetic knockdown of PINK1 or Parkin completely reversed the endothelial protective effect of FLNC, confirming that the protective effect of FLNC depended on functional triggering of the PINK1/Parkin mitophagy axis.

### 3.6. Proposed Molecular Mechanism of FLNC-Mediated Protection Against Atherosclerosis

**Figure 6:**
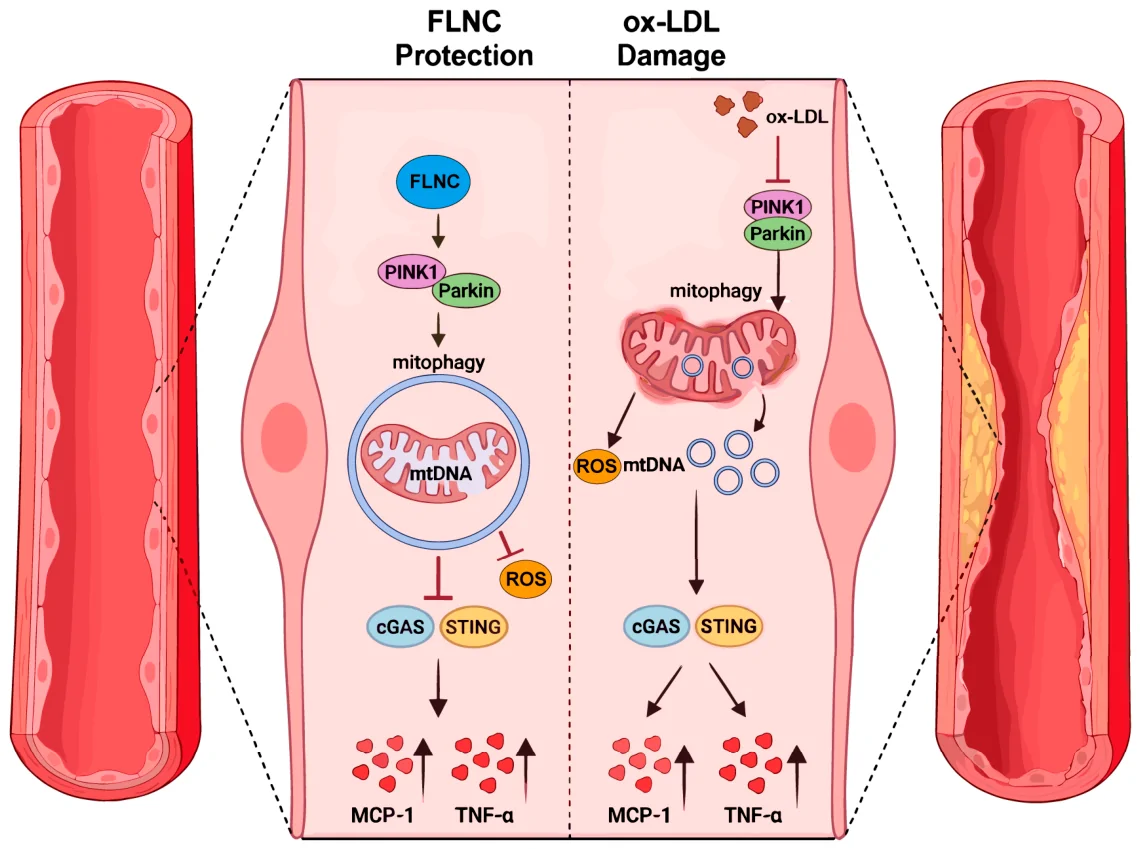
Schematic illustration of the protective mechanism of FLNC against atherosclerosis. FLNC stimulates PINK1/Parkin-dependent signaling and facilitates mitophagy, which selectively eliminates damaged mitochondria and inhibits mtDNA efflux to the cytosol. Diminished mtDNA accumulation within the cytosol hinders the initiation of the cGAS-STING immune signal transduction cascade, thereby attenuating endothelial cell apoptosis, oxidative stress, and inflammatory response, and ultimately alleviating the progression of atherosclerosis.

Collectively, our cellular and animal experimental data demonstrated that FLNC exerted a potent endothelial protective effect against atherosclerosis. Mechanistically, FLNC induces activation of the PINK1/Parkin mitophagy axis and facilitates mitochondrial clearance, which eliminates damaged mitochondria and inhibits mtDNA release into the cytoplasm. Suppression of cGAS-STING inflammatory axis activation arises from diminished cytoplasmic mtDNA, resulting in relieved endothelial apoptosis, oxidative stress and inflammatory damage. A schematic diagram depicting this protective signaling mechanism is shown in Figure 6.

## 4. Discussion

To our knowledge, this study is the first to characterize FLNC as a novel endogenous protective factor in vascular endothelial cells, with three core original findings. First, FLNC expression was markedly downregulated in atherosclerotic endothelial injury, and its deficiency directly promoted apoptosis and inflammatory activation of endothelial cells; meanwhile, endothelial dysfunction serves as a vital priming factor driving the onset and deterioration of atherosclerosis [6]. Second, FLNC directly targeted and regulated the PINK1/Parkin pathway, acting as a novel upstream activator of mitophagy in endothelial cells. Third, FLNC preserved the function of endothelial cells and exerted anti-atherosclerotic effects by enhancing mitophagy, thereby inhibiting the cytoplasmic efflux of mtDNA and suppressing downstream activation of the cGAS-STING innate immune signaling axis. These findings fundamentally challenge the conventional view that FLNC functions solely as a structural cytoskeletal protein in striated muscle [28], and fill a critical gap in the understanding of endogenous upstream regulators of endothelial mitophagy.

Previous studies on the function of FLNC have been largely restricted to cytoskeletal dynamic architectural reorganization and stabilization of intracellular physiological equilibrium across striated muscle, with a primary focus on the association between FLNC mutations and muscular dystrophies or cardiomyopathies. Notably, these investigations have entirely overlooked its potential non-canonical functions in vascular biology and non-muscle cell types. A key breakthrough of the present study is that FLNC acted far more than merely a structural cytoskeletal scaffold, but additionally serves as a vital upstream driver governing the mitophagy pathway, a central quality-control mechanism responsible for preserving mitochondrial homeostasis [29]. These findings align with recent advances demonstrating that actin-binding proteins modulate organelle trafficking and autophagosome formation [30], thereby expanding the functional landscape of cytoskeletal proteins in intracellular signal transduction. Of particular importance, we demonstrated for the first time that FLNC directly regulated the PINK1/Parkin pathway via facilitating the stable anchoring of PINK1 and Parkin onto the outer mitochondrial membrane, a rate-limiting step for the selective autophagic elimination of damaged mitochondria [31,32]. Furthermore, rescue experiments using siRNA-mediated silencing of PINK1 or Parkin entirely eliminated the endothelial protective effects of FLNC, providing definitive causal evidence that FLNC functioned through the PINK1/Parkin axis. The PINK1/Parkin cascade is widely recognized as the canonical mitophagy pathway and a key endothelial protective mechanism against atherosclerosis [14,33], and its activation reduces atherosclerotic lesion size in ApoE^−/−^ mice [34]. However, endogenous upstream regulators of this pathway in endothelial cells remain poorly defined. Moreover, no prior studies have functionally linked any member of the actin-binding protein family to this pathway. Our findings therefore directly fill this critical knowledge gap in the field.

The present study further established a complete molecular axis for FLNC-regulated endothelial inflammation, confirming that mitophagy served as the central intermediate step by which FLNC inhibited the cGAS-STING inflammatory pathway. Previous studies have demonstrated that impaired mitophagy results in the escape of mitochondrial DNA to the cytoplasm [35], whereas the cGAS-STING cascade activated by cytosolic mtDNA leakage constitutes a core mechanism driving chronic inflammation in atherosclerosis [15,36]. Sustained activation of this pathway in endothelial cells directly promotes monocyte adhesion, transendothelial migration, and atherosclerotic plaque progression [37,38]. Our results verified that FLNC inhibits cytosolic mtDNA release by enhancing mitophagy, thereby suppressing excessive triggering of the cGAS-STING innate immune axis as well as the consequent generation of pro-inflammatory factors including TNF-α and MCP-1. Notably, blockade of mitophagy or silencing of PINK1/Parkin completely abolished the anti-inflammatory effects of FLNC. These findings not only clarified the central mechanism underlying endothelial inflammatory cascades induced by FLNC deficiency, but also established a direct regulatory link between mitochondrial quality control and endothelial innate immune inflammation, providing a novel upstream intervention target for anti-atherosclerotic therapies targeting the cGAS-STING pathway. In addition, the eNOS and its Ser1177 phosphorylation status are important indicators of endothelial-dependent vasodilatory function and endothelial homeostasis [39,40]. Our supplementary experiments in both HUVECs and HAECs showed that FLNC did not alter total eNOS expression, but significantly regulated eNOS phosphorylation at Ser1177 (the activation status of eNOS). This result indicated that FLNC exerted endothelial protection at least partially by restoring eNOS activity rather than affecting its total expression, complementing the functional evidence of FLNC-mediated endothelial homeostasis maintenance.

The multiple verification strategies used in this study maximally ensured the reliability and translational value of our conclusions. In light of the controversy over potential off-target effects of the mitophagy inhibitor Mdivi-1 [41,42], pharmacological intervention results were not regarded as core causal evidence, but instead served as orthogonal complementary validation to genetic silencing of PINK1/Parkin. Highly consistent results obtained from these two independent intervention approaches thoroughly eliminated the limitations of single experimental methods, and firmly confirmed that the protective effects of FLNC depended entirely on PINK1/Parkin-mediated mitophagy. In vivo experiments were performed by delivering target genes to endothelial cells using an AAV9 vector driven by the endothelial-specific Tie2 promoter, thus avoiding non-specific interference caused by systemic overexpression. The strategy using the endothelial-specific Tie2 promoter has been widely adopted in studies of vascular endothelial cells [43,44,45]. Primary endothelial and smooth muscle cells derived from mouse aorta were isolated using the classic aortic lumen collagenase perfusion method [26], and cell purity was verified with Western blotting for CD31 and α-SMA. This provided a reliable experimental system for accurately dissecting the endothelial-specific functions of FLNC at the animal level. The in vivo results showed that endothelial-specific overexpression of FLNC significantly reduced aortic plaque area and lipid content in ApoE^−/−^ mice, providing direct functional evidence for the anti-atherosclerotic role of FLNC in vivo and laying a solid foundation for its translational application in vivo. Meanwhile, the finding that FLNC expression was significantly downregulated in human atherosclerotic plaques further linked our basic mechanistic discoveries to clinical pathological phenotypes, suggesting that FLNC represents a potential candidate target for endothelial-targeted therapy against atherosclerosis.

The present work possesses several inherent limitations that merit subsequent discussion and exploration. First, the specific molecular mechanism by which FLNC regulates the PINK1/Parkin pathway was not fully elucidated. Whether FLNC modulates the mitochondrial localization and protein stability of PINK1 through direct protein–protein interaction, or affects the initiation of mitophagy by regulating the spatial interaction between mitochondria and the cytoskeleton, remains to be further clarified using experiments such as co-immunoprecipitation and mass spectrometry. Second, the number of clinical samples in this study was limited. Extended clinical surveillance combined with enlarged patient cohorts will be essential to corroborate how FLNC expression correlates with patient prognosis following detrimental cardiovascular complications. Third, it should be noted that Mdivi-1, the pharmacological inhibitor used in this study, is a widely applied tool for inhibiting mitochondrial fission and mitophagy. Previous studies have demonstrated that the antioxidant effects of Mdivi-1 are at least partially independent of Drp1 GTPase activity, and may be mediated by upregulating antioxidant enzymes including SOD1 and catalase, as well as activating the Nrf2 signaling pathway [46]. In addition, Mdivi-1 acts as a reversible inhibitor of mitochondrial respiratory chain complex I, and its validity as a specific Drp1 inhibitor in mammalian cells has long been debated [47]. Its effects on cell survival and mitochondrial function may also vary across cell types and experimental conditions. To circumvent the limitations of pharmacological tools and establish a clear causal relationship, we employed siRNA-mediated knockdown of PINK1 and Parkin as a complementary genetic approach alongside Mdivi-1 treatment. The highly consistent results obtained from both pharmacological and genetic interventions strongly demonstrate that FLNC exerts endothelial protective effects through PINK1/Parkin-dependent mitophagy, rather than through the off-target effects of Mdivi-1. Fourth, although this study employed both human umbilical vein endothelial cells (HUVECs) and human aortic endothelial cell line (HAECs) to validate the core conclusions, inherent limitations of in vitro endothelial cell models remain. HUVECs are derived from venous endothelium, which exhibit intrinsic differences in transcriptomic profiles, metabolic characteristics, and responses to shear stress and oxidative stress compared with arterial endothelial cells—the primary cell type affected in atherosclerosis. Although HAEC validation partially improved the universality of our findings, two-dimensional cultured endothelial cells in vitro cannot fully recapitulate the complex three-dimensional microenvironment of arterial endothelium in vivo, including pulsatile blood flow shear stress, paracrine interactions with smooth muscle cells and immune cells, and native extracellular matrix components. Further validation using primary arterial endothelial cells, vascular organoids or microfluidic chips is warranted to confirm the in vivo relevance of our conclusions. Last, this study mainly focused on the functional impact of FLNC within vascular endothelial cells throughout atherosclerotic lesion development. Considering the progression of atherosclerotic lesions involves the coordinated action of multiple cell types [48], further exploration is needed regarding its regulatory functions in other cell types, including macrophages, lymphocytes and vascular smooth muscle cells, together with its potential roles in other endothelial injury-related vascular diseases such as aortic aneurysm [49].

This study is the first to uncover the protective function and underlying molecular mechanism of FLNC in vascular endothelial cells. We demonstrated that FLNC inhibits the mtDNA-cGAS-STING inflammatory pathway via the PINK1/Parkin-mitophagy axis, thereby attenuating atherosclerosis progression. This finding not only enriches the molecular regulatory network governing endothelial injury in atherosclerosis but also breaks through the traditional functional paradigm of FLNC, providing a novel theoretical basis and potential candidate targets to facilitate the endothelial-targeted treatment and prevention of atherosclerosis.

## Data Availability

The data supporting this study are available from the corresponding authors upon reasonable request.

## Supplementary Materials

The following supporting information can be downloaded at: https://www.mdpi.com/article/10.3390/jcdd13090428/s1, Figure S1: FLNC is downregulated in atherosclerotic models and endothelial cell-specific overexpression of FLNC attenuates atherosclerotic plaque progression in ApoE^−/−^ mice; Figure S2: FLNC activates PINK1/Parkin-mediated mitophagy in HUVECs; Figure S3: FLNC activates PINK1/Parkin-mediated mitophagy in HAECs; Figure S4: FLNC inhibits ox-LDL-induced apoptosis and oxidative stress in HUVECs, suppresses mtDNA release and thereby blocks the cGAS-STING inflammatory pathway activation; Figure S5: FLNC inhibits ox-LDL-induced apoptosis and oxidative stress in HAECs, suppresses mtDNA release and thereby blocks the cGAS-STING inflammatory pathway activation; Figure S6: FLNC exerts its protective effects in a mitophagy-dependent manner in HUVECs; Figure S7: FLNC exerts its protective effects via the PINK1/Parkin signaling pathway in HUVECs; Figure S8: FLNC exerts its protective effects via the PINK1/Parkin signaling pathway in HAECs.

## Abbreviations

The following abbreviations are used in this manuscript:

α-SMA	Alpha-Smooth Muscle Actin
AAV9	Adeno-Associated Virus 9
ANOVA	Analysis of Variance
ApoE^−/−^	Apolipoprotein E knockout
AS	Atherosclerosis
BCA	Bicinchoninic Acid
BSA	Bovine Serum Albumin
cGAS	Cyclic GMP-AMP Synthase
CCCP	Carbonyl cyanide 3-chlorophenylhydrazone
CD31	Cluster of Differentiation 31
CRISPRa	Clustered Regularly Interspaced Short Palindromic Repeats Activation
CRISPRi	Clustered Regularly Interspaced Short Palindromic Repeats Interference
DAPI	4′,6-diamidino-2-phenylindole
DMSO	Dimethyl Sulfoxide
ECL	Enhanced Chemiluminescence
FLNC	Filamin C
H&E	Hematoxylin and Eosin
HFD	High-Fat Diet
HSD	Honestly Significant Difference
HUVEC	Human Umbilical Vein Endothelial Cell
IRF3	Interferon Regulatory Factor 3
JC-1	5,5′,6,6′-tetrachloro-1,1′,3,3′-tetraethylbenzimidazolylcarbocyanine iodide
LAMP1	Lysosome-Associated Membrane Protein 1
LC3	Microtubule-associated protein 1 light chain 3
MCP-1	Monocyte Chemoattractant Protein-1
Mdivi-1	Mitochondrial division inhibitor 1
mtDNA	Mitochondrial DNA
mtROS	Mitochondrial Reactive Oxygen Species
NCD	Normal Chow Diet
p62	Sequestosome 1
PAEC	Primary Aortic Endothelial Cell
Parkin	Parkin RBR E3 ubiquitin protein ligase
PASMC	Primary Aortic Smooth Muscle Cell
PFA	Paraformaldehyde
PINK1	PTEN-induced putative kinase 1
PVDF	Polyvinylidene Difluoride
RIPA	Radioimmunoprecipitation Assay
ROS	Reactive Oxygen Species
SDS-PAGE	Sodium Dodecyl Sulfate-Polyacrylamide Gel Electrophoresis
SEM	Standard Error of the Mean
SPF	Specific Pathogen Free
STING	Stimulator of Interferon Genes
TBK1	TANK-binding kinase 1
TEM	Transmission Electron Microscopy
TFAM	Mitochondrial Transcription Factor A
TOM20	Translocase of Outer Mitochondrial Membrane 20
TNF-α	Tumor Necrosis Factor-alpha
TUNEL	Terminal deoxynucleotidyl transferase dUTP nick end labeling
WB	Western Blotting
