# Supplementary material for: Endothelial Filamin C Alleviates Atherosclerosis via PINK1/Parkin-Dependent Mitophagy and mtDNA-cGAS-STING Inflammation Suppression
Source: J Cardiovasc Dev Dis. 2026 Sep 1;13(9):428. doi: 10.3390/jcdd13090428 (PMC13607726; doi:10.3390/jcdd13090428)
Supplement: Supplementary file 1 [file jcdd-13-00428-s001.zip › jcdd-4451868-supplementary.pdf]

Endothelial Filamin C Alleviates Atherosclerosis via PINK1/Parkin-Dependent Mitophagy and mtDNA-cGAS-STING Inflammation Suppression

Zhan et al.

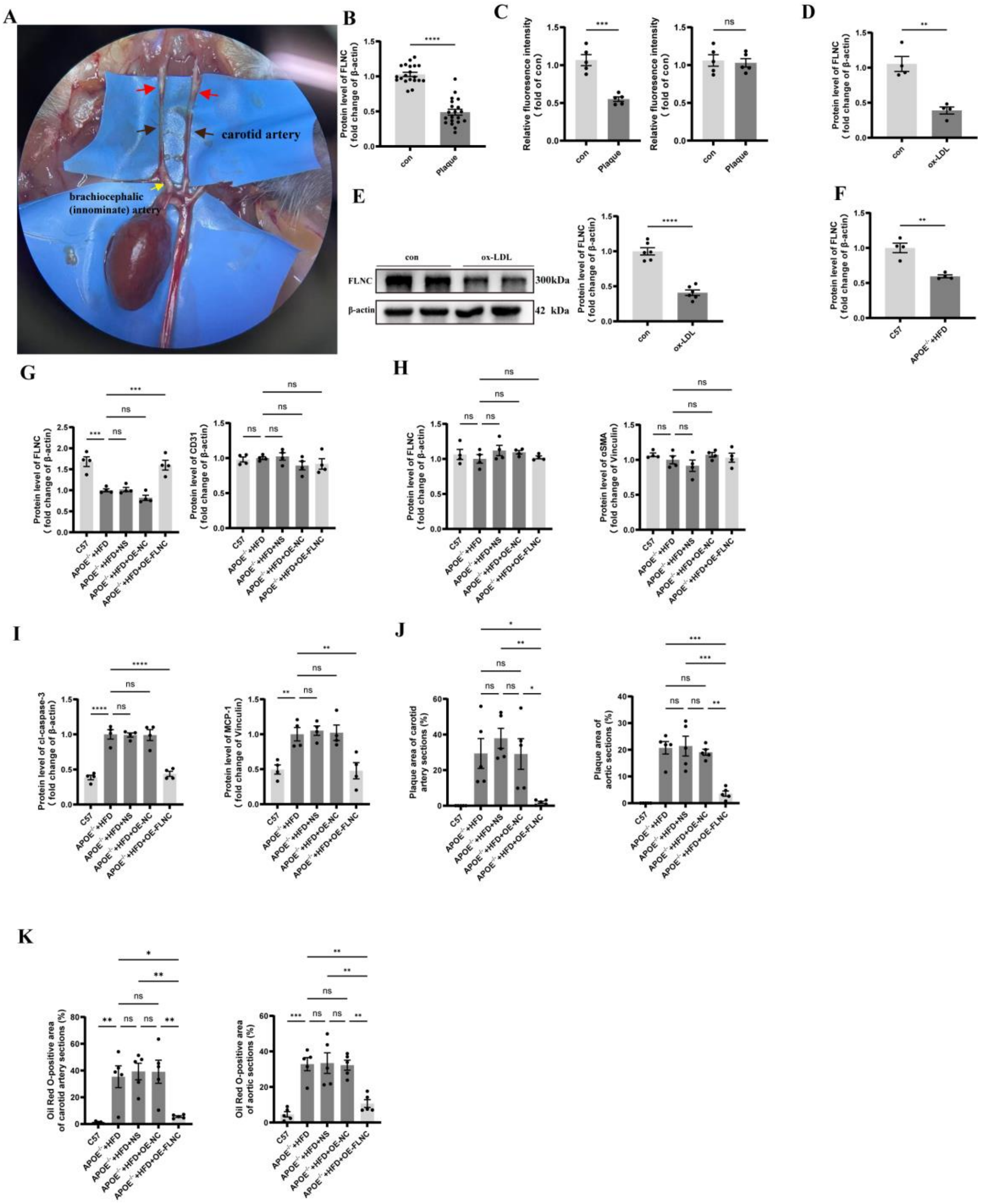

Fig. S1 FLNC is downregulated in atherosclerotic models, and endothelial cell-specific overexpression of FLNC attenuates atherosclerotic plaque progression in ApoE$^{-/-}$ mice. FLNC protein expression levels were detected in clinical samples, in vitro cell models, and in vivo animal models. We then validated the purity of isolated primary endothelial cells, confirmed the endothelial expression of FLNC in mouse aortas, and evaluated the functional effects of endothelial FLNC overexpression on endothelial apoptosis, inflammation and atherosclerotic plaque formation in ApoE$^{-/-}$ mice. (A) Schematic diagram of the sampling site at the mouse carotid artery bifurcation. Yellow arrow: brachiocephalic trunk; Red arrow: carotid artery bifurcation; Black arrow: common carotid artery. (B) Western blotting analysis of FLNC expression in human carotid atherosclerotic plaques and paired adjacent normal vascular tissues (n=20). (C) Quantitative analysis of FLNC fluorescence intensity in CD31-positive endothelial regions and α-SMA-positive smooth muscle regions (n=5). (D) Western blotting analysis of FLNC expression in HUVECs treated with 25 μg/mL ox-LDL for 24 h (n=4). (E) Western blotting analysis of FLNC expression in HUVECs treated with 25 μg/mL ox-LDL for 24 h (n=4). (F) Western blotting analysis of FLNC expression in primary aortic endothelial cells isolated from normal chow diet (NCD)-fed C57BL/6J mice and high-fat diet

(HFD)-fed ApoE$^{-/-}$ mice (n=4). (G) Western blotting analysis of FLNC, CD31 and α-SMA (smooth muscle cell marker) expression in primary aortic endothelial cells (n=4). No detectable α-SMA bands were observed, confirming high purity of isolated endothelial cells. (H) Western blotting analysis of FLNC, α-SMA and CD31 (endothelial cell marker) expression in primary aortic smooth muscle cells (n=4). No detectable CD31 bands were observed, confirming high purity of isolated smooth muscle cells. (I) Western blotting analysis of cleaved-caspase-3 and MCP-1 expression in primary aortic endothelial cells (n=4). (J) Histological analysis of atherosclerotic plaques by H&E staining. Plaque areas in both carotid arteries and aortas were quantified; scale bar: 50 μm for carotid artery, 200 μm for aorta (n=5). (K) Lipid content analysis by Oil Red O staining. Lipid contents in both carotid arteries and aortas were quantified; scale bar: 50 μm for carotid artery, 200 μm for aorta (n=5). Data are presented as mean ± SEM. ns non-significant, *P < 0.05, **P < 0.01, ***P < 0.001, ****P < 0.0001.

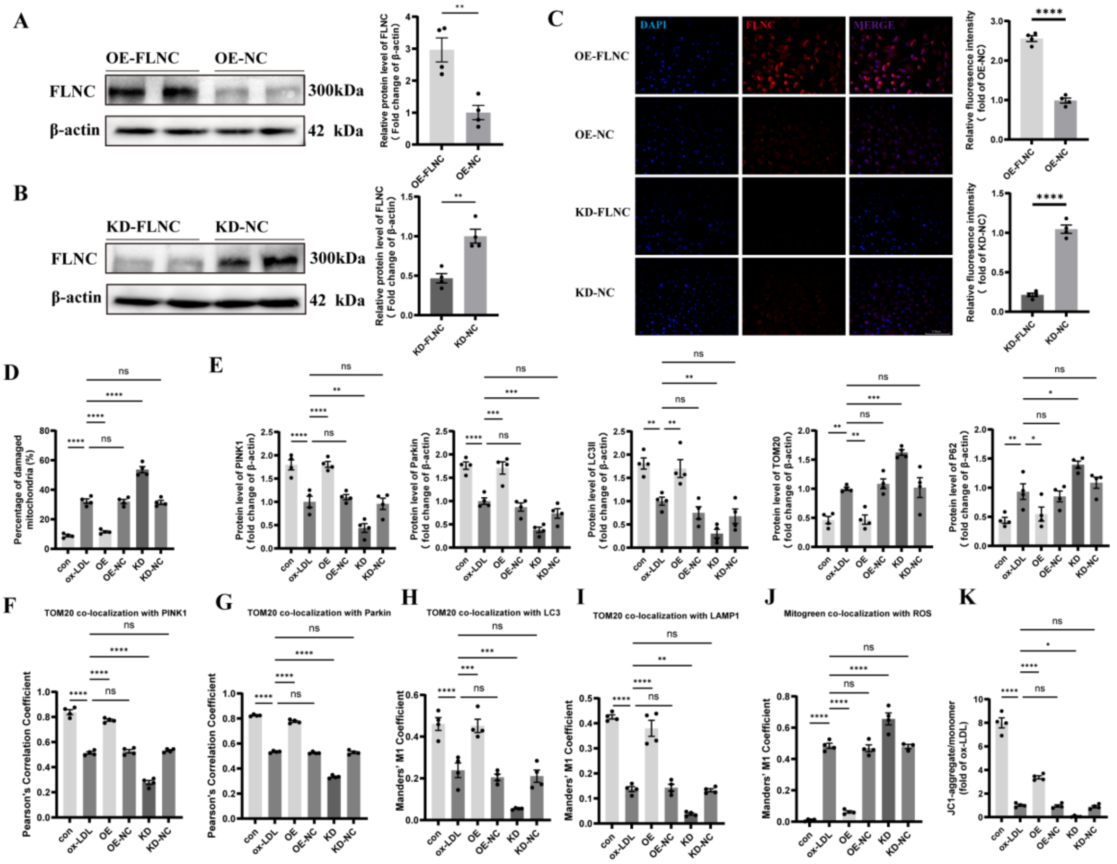

Fig. S2 FLNC activates PINK1/Parkin-mediated mitophagy in HUVECs. Stable HUVEC lines with FLNC overexpression (OE) or knockdown (KD) were established using CRISPRa and CRISPRi systems, respectively and the effect of FLNC on PINK1/Parkin-mediated mitophagy was evaluated. (A-B) Western blotting analysis was used to verify FLNC expression levels (n=4). (C) Immunofluorescence staining for FLNC was performed to confirm protein expression changes (scale bar: 100 μm, n=4,). (D) Quantitative analysis of the percentage of damaged mitochondria under transmission electron microscopy (TEM) in HUVECs. (E) Western blotting analysis of mitophagy-related proteins (PINK1, Parkin, LC3II, p62, and TOM20) (n=4). (F-G) Co-immunofluorescence staining of TOM20 with PINK1 or Parkin; co-localization was quantified using Pearson correlation coefficient (scale bar: 17 μm, n=4,). (H-I) Co-immunofluorescence staining of TOM20 with LC3 (autophagosome marker) or LAMP1 (lysosome marker); co-localization was quantified using Manders overlap coefficient (scale bar: 17 μm, n=4,). (J) Mitochondrial ROS (mtROS) levels were measured by MitoSOX staining (scale bar: 200 μm, n=4). (K) Mitochondrial membrane potential was detected by JC-1 staining (scale bar: 200 μm, n=4). Data are presented as mean ± SEM. ns non-significant, *P < 0.05, **P < 0.01, ***P < 0.001, ****P < 0.0001.

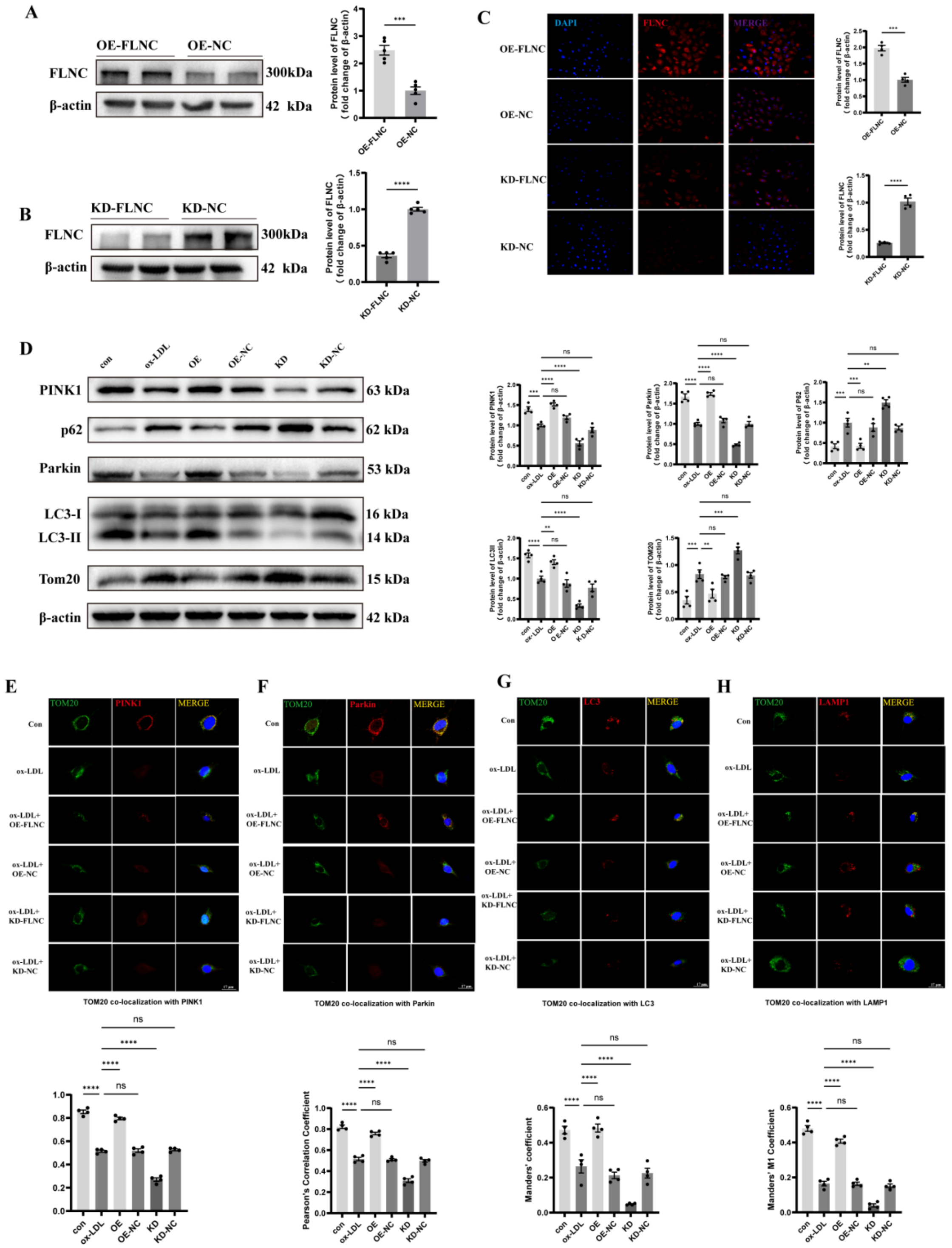

Fig. S3 FLNC activates PINK1/Parkin-mediated mitophagy in HAECs. Stable HAEC lines with FLNC overexpression (OE) or knockdown (KD) were established using CRISPRa and CRISPRi systems, respectively and the effect of FLNC on PINK1/Parkin-mediated mitophagy was evaluated. (A-B) Western blotting analysis was used to verify FLNC expression levels (n=4). (C) Immunofluorescence staining for FLNC was performed to confirm protein expression changes (n=4, scale bar: 100 μm). (D) Western blotting analysis of mitophagy-related proteins (PINK1, Parkin, LC3II, p62, and TOM20) (n=4). (E-F) Co-immunofluorescence staining of TOM20 with PINK1 or Parkin; co-localization was quantified using Pearson correlation coefficient (scale bar: 17 μm, n=4,). (J-H) Co-immunofluorescence staining of TOM20 with LC3 (autophagosome marker) or LAMP1 (lysosome marker); co-localization was quantified using Manders overlap coefficient (scale bar: 17 μm, n=4,). Data are presented as mean ± SEM. ns non-significant, *$P < 0.05$,

$^{**}P < 0.01$, $^{***}P < 0.001$, $^{****}P < 0.0001$.

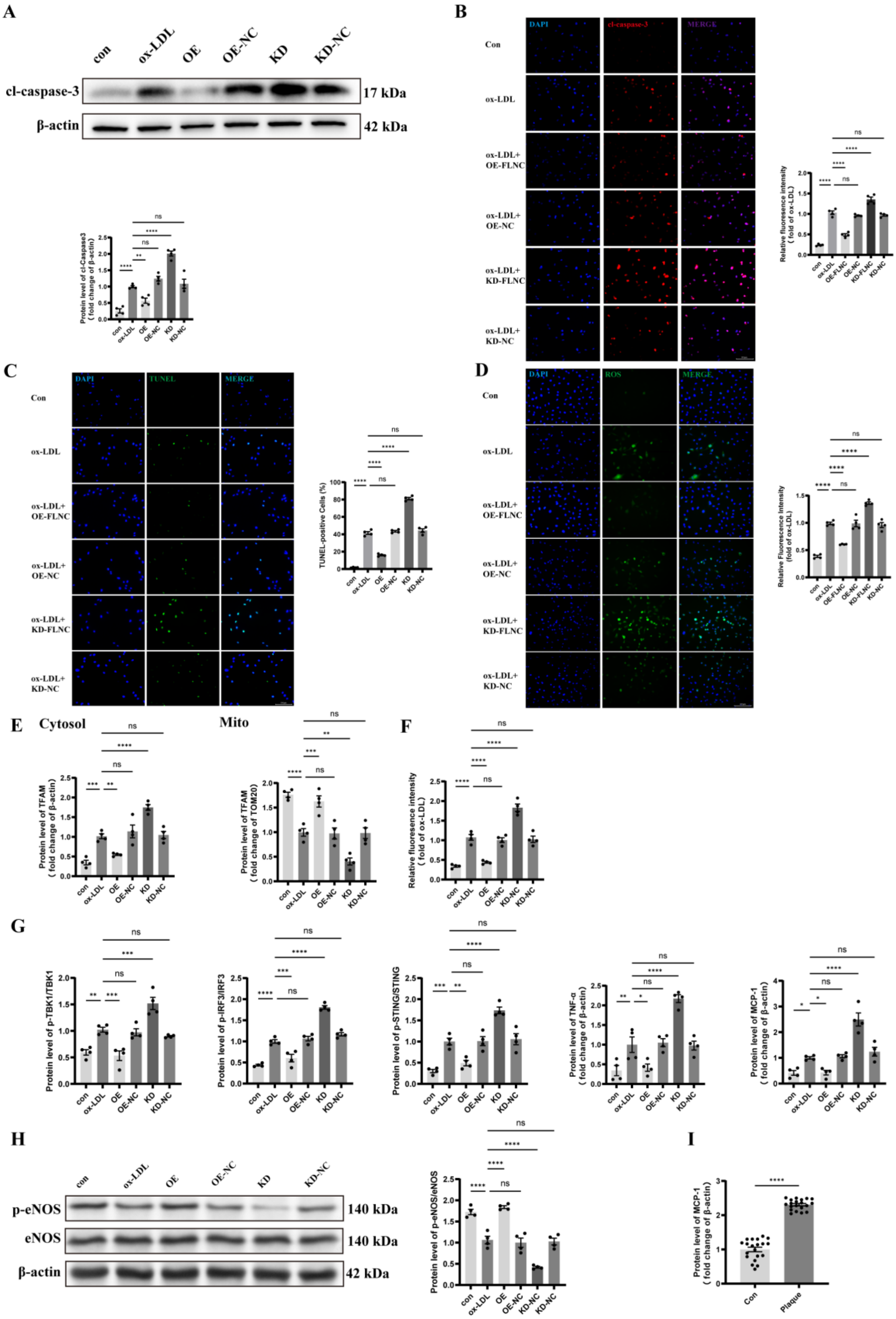

Fig. S4 FLNC inhibits ox-LDL-induced apoptosis and oxidative stress in HUVECs, suppresses mtDNA release and thereby blocks

the cGAS-STING inflammatory pathway activation. The effect of FLNC on apoptosis and mtDNA release and cGAS-STING pathway activation was investigated. (A) Western blot analysis of cleaved-caspase-3 expression (n=4). (B) Immunofluorescence staining for cleaved-caspase-3 (scale bar: 100 µm, n=4). (C) TUNEL assay was used to detect endothelial cell apoptosis (scale bar: 100 µm, n=4). (D) Intracellular ROS levels were measured by DCFH-DA staining (scale bar: 100 µm, n=4). (E) Subcellular fractionation followed by Western blotting was performed to detect TFAM levels in cytoplasmic and mitochondrial fractions (n=4). The purity of the isolated fractions was confirmed by the absence of TOM20 in cytoplasmic extracts and β-actin in mitochondrial preparations. (F) Quantification of cytosolic PicoGreen fluorescence intensity in HUVECs (n=4). (G) Western blot analysis of cGAS-STING pathway components and downstream pro-inflammatory cytokines (n=4). (H) Western blotting analysis of total eNOS and p-eNOS (Ser1177) levels in HUVECs (n=4). (I) Western blot analysis of MCP-1 expression in human carotid atherosclerotic plaques and paired adjacent normal vascular tissues (n=20). Data are presented as mean ± SEM. ns non-significant, *$P < 0.05$, **$P < 0.01$, ***$P < 0.001$, ****$P < 0.0001$. Phosphorylated and total protein levels were detected on the same blots after membrane stripping with Western Stripping buffer.

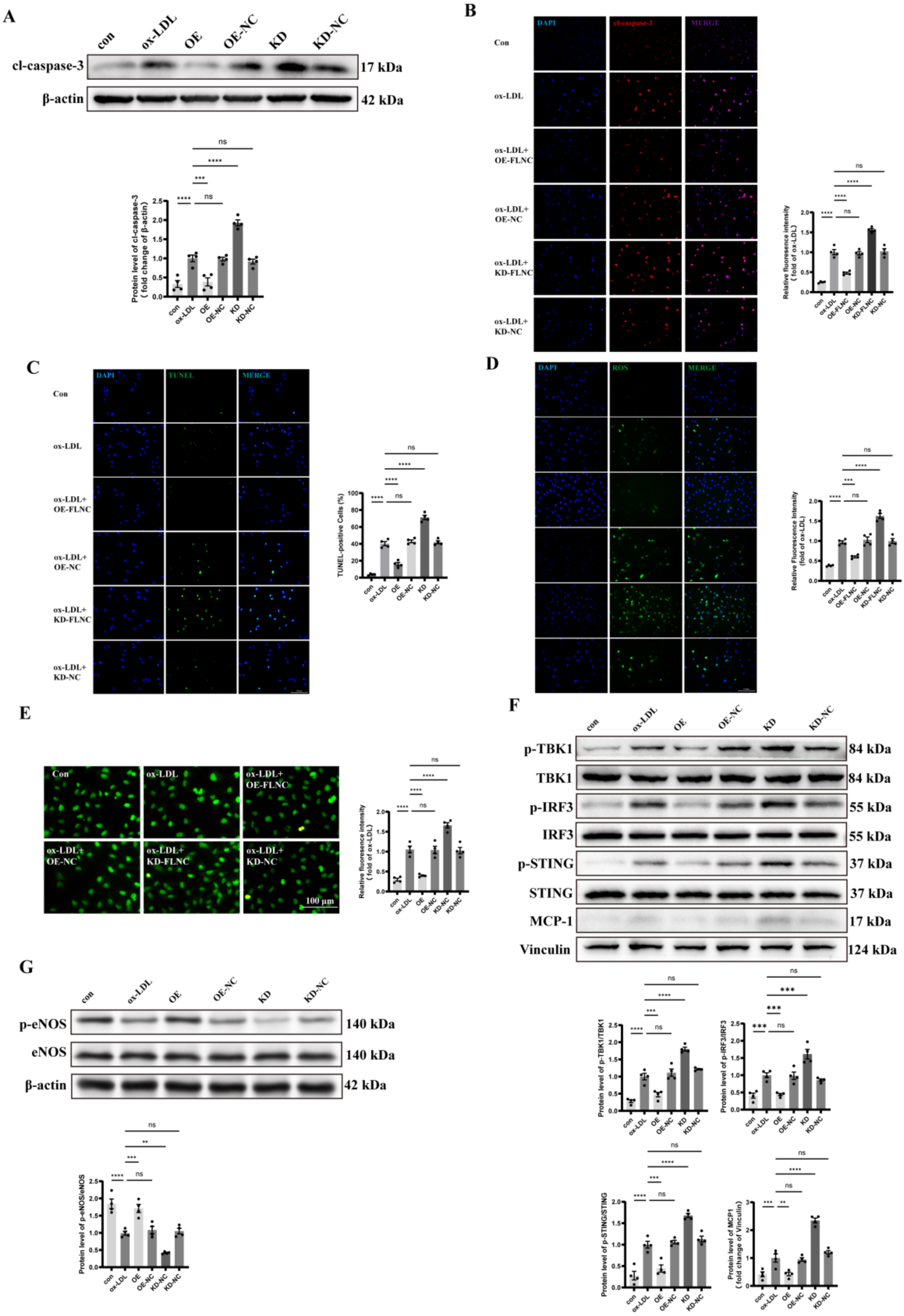

Fig. S5 FLNC inhibits ox-LDL-induced apoptosis and oxidative stress in HAECs, suppresses mtDNA release and thereby blocks the cGAS-STING inflammatory pathway activation. The effect of FLNC on apoptosis and mtDNA release and cGAS-STING pathway activation was investigated. (A) Western blot analysis of cleaved-caspase-3 expression (n=4). (B) Immunofluorescence staining for cleaved-caspase-3 (scale bar: 100 μm, n=4). (C) TUNEL assay was used to detect endothelial cell apoptosis (scale bar: 100 μm, n=4). (D) Intracellular ROS levels were measured by DCFH-DA staining (scale bar: 100 μm, n=4). (E) Quantification of cytosolic PicoGreen

fluorescence intensity in HAECs (scale bar: 100 μm, n=4). (F) Western blot analysis of cGAS-STING pathway components and downstream pro-inflammatory cytokines (n=4). (G) Western blotting analysis of total eNOS and p-eNOS (Ser1177) levels in HAECs (n=4). Data are presented as mean ± SEM. ns non-significant, **P* < 0.05, ***P* < 0.01, ****P* < 0.001, *****P* < 0.0001. Phosphorylated and total protein levels were detected on the same blots after membrane stripping with Western Stripping buffer.

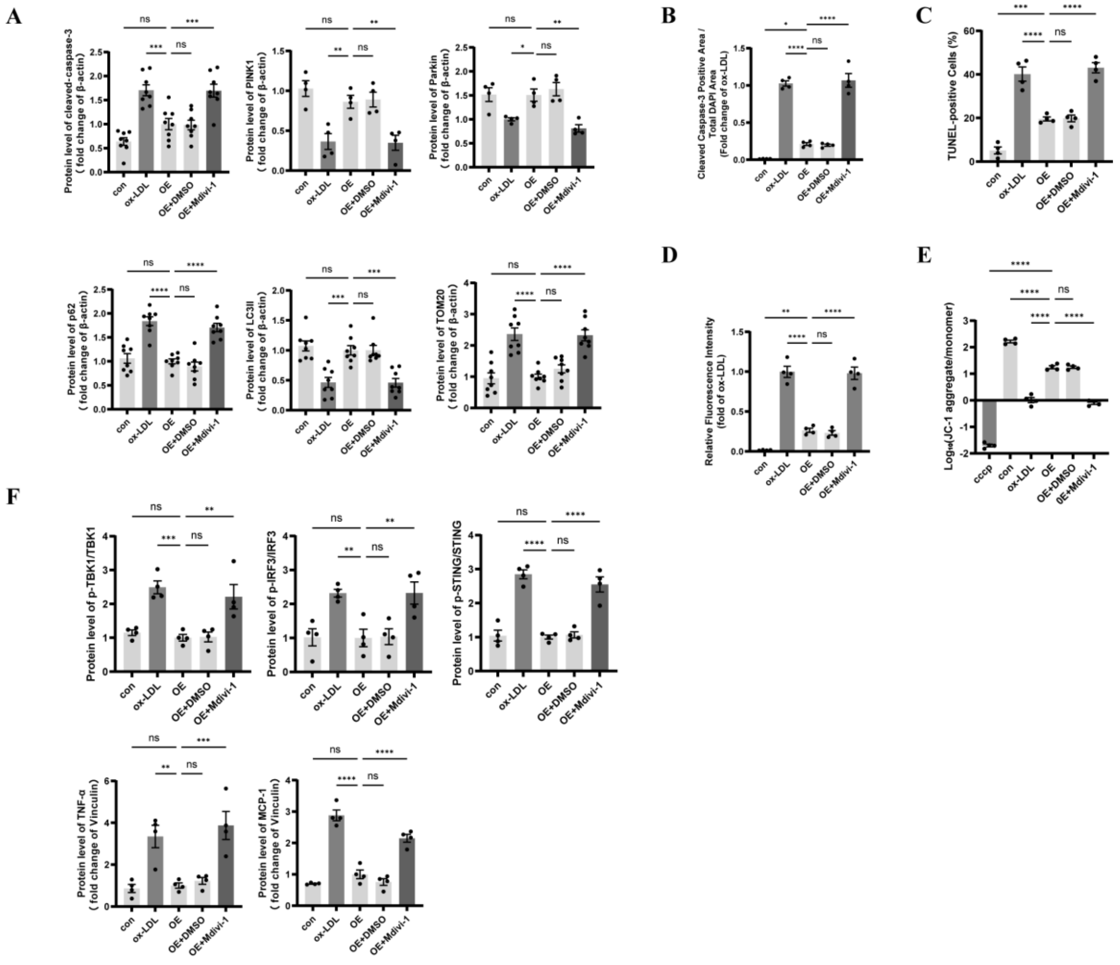

Fig. S6 FLNC exerts its protective effects in a mitophagy-dependent manner in HUVECs. Rescue experiments were performed using Mdivi-1, a pharmacological inhibitor of mitophagy. (A) Western blotting analysis of mitophagy-related proteins and cleaved-caspase-3. PINK1 and Parkin (n=4); LC3II, p62, TOM20, and cleaved-caspase-3 (n=8). (B) Immunofluorescence staining for cleaved-caspase-3 (scale bar: 100 μm, n=4). (C) TUNEL assay was used to detect endothelial cell apoptosis (scale bar: 100 μm, n=4). (D) Intracellular ROS levels were measured by DCFH-DA staining (scale bar: 200 μm, n=4). (E) Mitochondrial membrane potential was detected by JC-1 staining (scale bar: 100 μm, n=4). (F) Western blotting analysis of cGAS-STING pathway components and downstream pro-inflammatory cytokines (n=4). Data are presented as mean ± SEM. ns non-significant, **P* < 0.05, ***P* < 0.01, ****P* < 0.001, *****P* < 0.0001. Phosphorylated and total protein levels were detected on the same blots after membrane stripping with Western Stripping buffer.

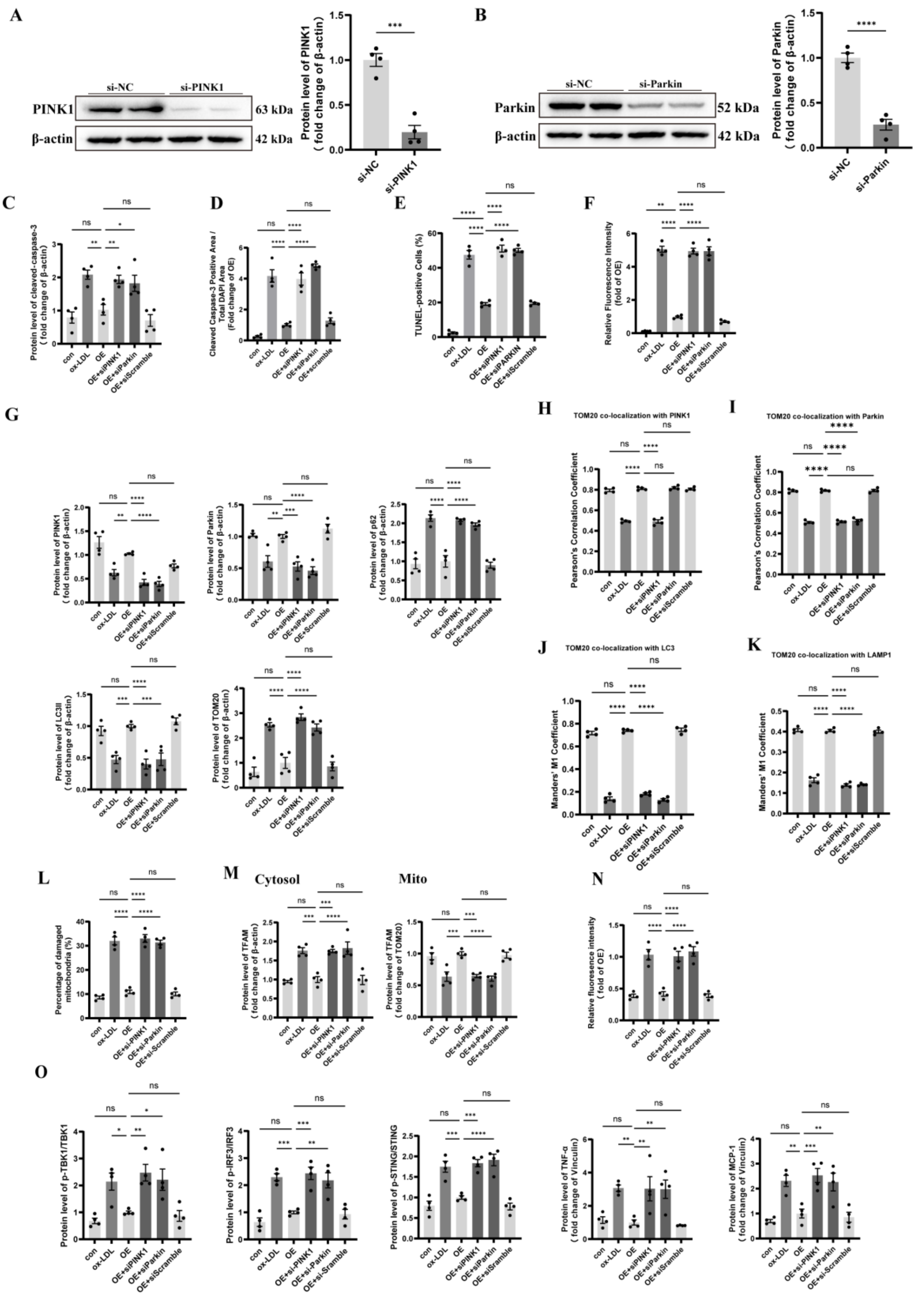

Fig. S7 FLNC exerts its protective effects via the PINK1/Parkin signaling pathway in HUVECs. Rescue experiments were performed using siRNA-mediated knockdown of PINK1 or Parkin. (A-B) Western blotting analysis was performed to verify the knockdown efficiency of si-PINK1 and si-Parkin (n=4). (C) Western blotting analysis of cleaved-caspase-3 expression (n=4). (D) Immunofluorescence staining for cleaved-caspase-3 (scale bar: 100μm, n=4). (E) TUNEL assay was used to detect endothelial cell apoptosis (scale bar: 100 μm, n=4). (F) Intracellular ROS levels were measured by DCFH-DA staining (scale bar: 200 μm, n=4). (G) Western blotting analysis of mitophagy-related proteins (n=4). (H-I) Co-immunofluorescence staining of TOM20 with PINK1 and Parkin;

co-localization was quantified using Pearson correlation coefficient (scale bar: 17 μm, n=4). (J-K) Co-immunofluorescence staining of TOM20 with LC3 (autophagosome marker) and LAMP1 (lysosome marker); co-localization was quantified using Manders overlap coefficient (scale bar: 17 μm, n=4). (L) Quantification of damaged mitochondrial percentage under TEM in HUVECs (n=4). (M) Subcellular fractionation followed by Western blotting was performed to detect cytoplasmic TFAM levels (n=4). The purity of the isolated fractions was confirmed by the absence of TOM20 in cytoplasmic extracts and β-actin in mitochondrial preparations. (N) Quantification of cytosolic PicoGreen fluorescence intensity in HUVECs (n=4). (O) Western blotting analysis of cGAS-STING pathway components and downstream pro-inflammatory cytokines (n=4). Data are presented as mean ± SEM. ns non-significant, **P* < 0.05, ***P* < 0.01, ****P* < 0.001, *****P* < 0.0001. Phosphorylated and total protein levels were detected on the same blots after membrane stripping with Western Stripping buffer.

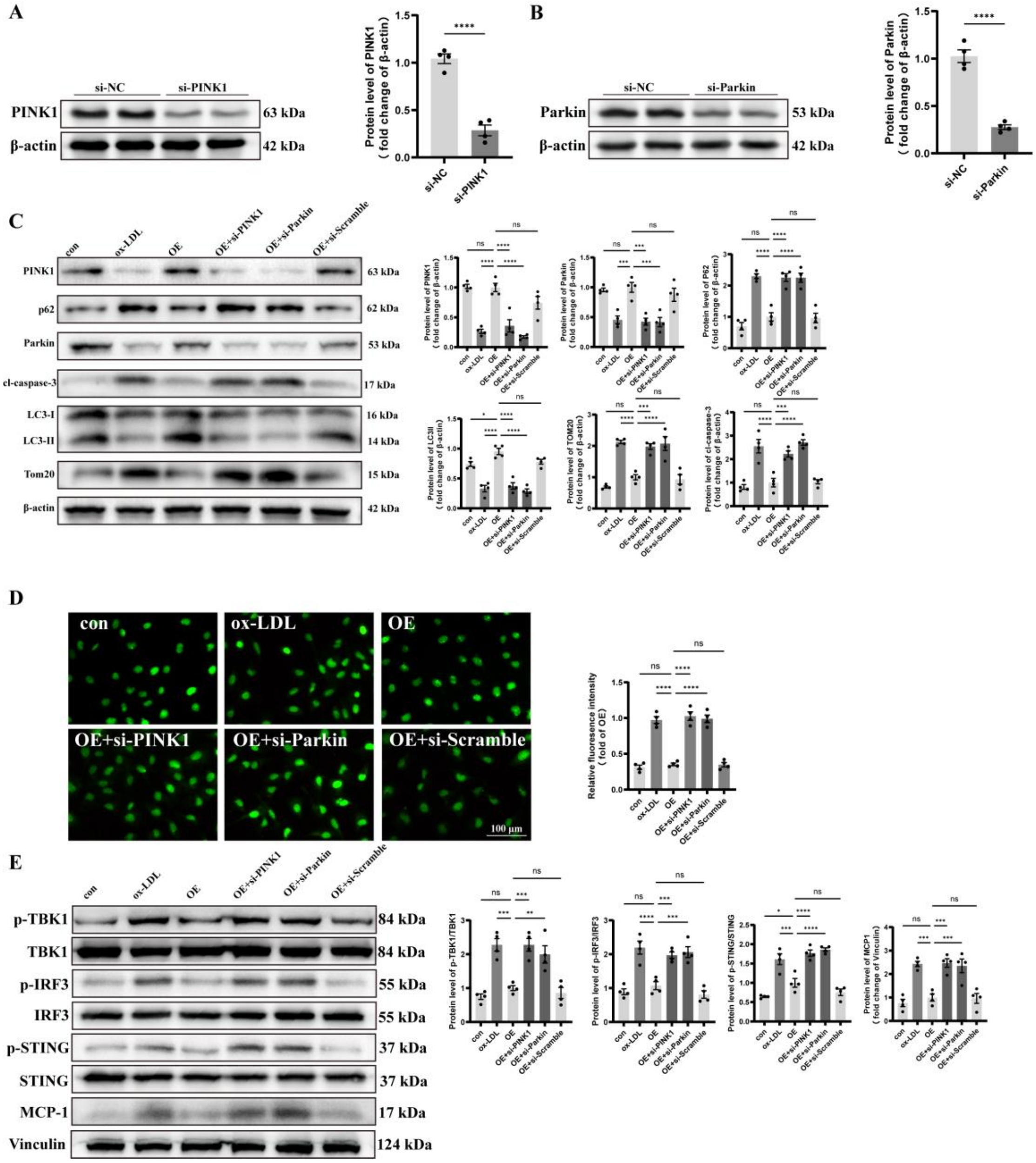

Fig. S8 FLNC exerts its protective effects via the PINK1/Parkin signaling pathway in HAECs. Rescue experiments were performed using siRNA-mediated knockdown of PINK1 or Parkin. (A-B) Western blotting analysis was performed to verify the knockdown efficiency of si-PINK1 and si-Parkin (n=4). (C) Western blotting analysis of cleaved-caspase-3 and mitophagy-related proteins expression (n=4). (D) Quantification of cytosolic PicoGreen fluorescence intensity in HAECs (scale bar: 100 μm, n=4). (E) Western blotting analysis of cGAS-STING pathway components and downstream pro-inflammatory cytokines (n=4). Data are presented as mean ± SEM. ns

non-significant, *$P < 0.05$, **$P < 0.01$, ***$P < 0.001$, ****$P < 0.0001$. Phosphorylated and total protein levels were detected on the same blots after membrane stripping with Western Stripping buffer.
